# Recent Advances in Polypyrrole-Based Functional Coatings: Surface Protection and Emerging Applications

**DOI:** 10.3390/ma19112213

**Published:** 2026-05-24

**Authors:** Ge Cao, Qiuyuan Huang, Yueying Li, Zhenyu Wang, En-Hou Han

**Affiliations:** 1 School of Textile, Guangdong Polytechnic, Gaoming, Foshan 528000, China; 2 Institute of Corrosion Science and Technology, Guangzhou 510530, China; msqiuyuanhuang@mail.scut.edu.cn (Q.H.); yyli@icost.ac.cn (Y.L.); 3 School of Materials Science and Engineering, South China University of Technology, Guangzhou 510641, China; 4 Guangdong Hetong Technology Co., Ltd., Dongguan 523808, China

**Keywords:** polypyrrole, functional coatings, surface protection, corrosion protection, conductive polymers, composite coatings, emerging applications

## Abstract

Owing to its inherent electrical conductivity, reversible redox activity, and structural versatility, polypyrrole (PPy) has become an important material for advanced functional coatings. This review summarizes recent advances in PPy-based coatings, systematically exploring the correlation between fundamental material design and macroscopic multifunctional applications. First, the core structural characteristics of PPy and its primary fabrication strategies, including electrochemical deposition, chemical oxidative polymerization, solution processing, and hybrid composite engineering, are delineated. Subsequently, the role of PPy in surface protection is analyzed, with an emphasis on the synergistic mechanisms underlying corrosion mitigation, mechanical durability, and environmental barriers (e.g., anti-fouling and solar-driven desalination). In addition, the application expansion of PPy in emerging fields, such as electromagnetic interference (EMI) shielding, highly sensitive smart sensing, electroactive energy interfaces, and advanced biomedical electrodes, is summarized. Finally, current challenges—particularly the physicochemical trade-offs among conductivity, interfacial adhesion, and long-term stability—are discussed, and future development directions are prospected. By integrating green processing technologies and data-driven smart system integration, next-generation PPy coatings are expected to meet the demands of flexible electronics, sustainable energy, and precision medicine.

## 1. Introduction

Functional coatings have become an important class of surface-engineered materials for extending service life, improving environmental resistance, and introducing advanced interfacial functions to structural and electronic substrates [1,2]. In modern industrial systems, surfaces are often exposed to corrosive media, mechanical wear, moisture, ultraviolet irradiation, and other complex service conditions that can rapidly deteriorate material performance [3,4]. Conventional protective coatings, such as organic barrier layers, metallic coatings, and ceramic films, have been widely used to mitigate these issues. However, many traditional coating systems still face persistent challenges, including limited electrical functionality, poor active protection capability, inadequate adaptability to multifunctional requirements, and difficulty in simultaneously achieving surface protection and emerging smart functions [5,6]. As a result, the development of multifunctional coatings that integrate protection, conductivity, and responsiveness has attracted increasing attention in recent years.

Intrinsically conductive polymers (ICPs) represent a unique class of organic materials that combine the processability of polymers with the electrical and electrochemical characteristics of conjugated systems [7,8]. Among them, polypyrrole (PPy) has been extensively investigated because of its relatively facile synthesis, tunable conductivity, redox activity, and diverse microstructural forms. PPy can be prepared through electrochemical deposition, chemical oxidative polymerization, interfacial polymerization, and various solution-based methods, making it suitable for constructing coatings on metals, polymers, textiles, and other substrates. In addition, the chemical structure of PPy allows it to be readily combined with resins, nanocarbons, metal oxides, two-dimensional materials, and other functional components, thereby offering broad opportunities for the design of hybrid and multifunctional coating systems [9,10].

Unlike conventional passive coatings, the particular advantage of PPy lies in its ability to function as an active interfacial material rather than only as a physical barrier [1,2]. Conventional organic coatings mainly protect substrates by blocking the transport of water, oxygen, and aggressive ions, whereas metallic and ceramic coatings usually rely on dense shielding or sacrificial protection [4,5]. In contrast, PPy combines electronic conductivity, reversible redox activity, and ion-coupled doping/dedoping behavior within one polymeric coating framework. These features allow PPy-based coatings to simultaneously provide conductive pathways, electrochemical passivation, interfacial charge regulation, and stimulus-responsive behavior [7,8,9,11]. Moreover, PPy can be formed directly on metals, polymers, textiles, and porous substrates through electrochemical deposition or chemical oxidative polymerization, enabling conformal coating on complex surfaces [12]. Therefore, the key competitiveness of PPy as a coating material is not a single high conductivity value, but the integration of conductivity, redox activity, structural tunability, substrate adaptability, and multifunctional interfacial response.

PPy-based coatings are particularly attractive for surface protection applications. Unlike conventional passive barrier coatings that mainly rely on blocking the penetration of water, oxygen, or ions, PPy-based systems may provide multiple protection pathways. These include physical barrier effects, interfacial passivation, redox-mediated protection, and synergistic enhancement in composite architectures [13]. Such features make PPy a promising candidate for corrosion-resistant coatings on steel, aluminum alloys, magnesium alloys, and other metallic substrates. At the same time, the intrinsic conductivity and electroactivity of PPy enable coating functions beyond traditional protection. By tailoring morphology, dopants, and composite structures, PPy-based coatings can also serve as antistatic layers, electromagnetic shielding coatings, sensing interfaces, electroactive surfaces, and energy-related functional films. This dual role of surface protection and functional integration distinguishes PPy-based coatings from many conventional protective materials.

Despite these advantages, several limitations still hinder the practical deployment of PPy-based coatings. Pure PPy coatings often suffer from brittleness, limited mechanical robustness, and insufficient long-term stability under harsh environments. Their adhesion to different substrates may vary significantly depending on deposition route and surface pretreatment. In addition, the conductivity, microstructure, and protective performance of PPy are strongly influenced by synthesis conditions, oxidants, dopants, counterions, and post-treatment processes [14,15]. These factors complicate structure–property optimization but also provide important opportunities for rational materials design. In recent years, increasing efforts have been devoted to overcoming these drawbacks through nanostructure regulation, interface engineering, composite design, and environmentally friendly fabrication strategies. As a result, a large body of literature has emerged on PPy-based coatings with improved durability, multifunctionality, and application-specific performance.

In parallel with advances in materials design, the application scope of PPy-based coatings has continued to expand. In addition to corrosion protection and barrier coatings, recent studies have demonstrated their utility in smart sensing surfaces, flexible conductive interfaces, wearable electronic coatings, electromagnetic interference shielding layers, electrochromic systems, and biofunctional interfaces [16,17]. This trend reflects a broader transition from single-function protective coatings to integrated surface systems capable of protection, signal response, energy interaction, or environmental adaptability. Therefore, a focused review on PPy-based functional coatings is both timely and necessary, especially from the perspective of connecting preparation methods, microstructural design, surface protection mechanisms, and emerging applications.

Taken together, the recent progress of PPy-based coatings suggests a clear transition from single-function conductive or protective films toward active interfacial systems with integrated protective and responsive functions. Therefore, this review emphasizes representative coating scenarios in which the intrinsic properties of PPy are directly translated into macroscopic performance. Surface protection is regarded as the primary focus, as it reflects the combined effects of barrier reinforcement, redox-mediated passivation, and interfacial regulation. Representative emerging applications, including antistatic and electromagnetic interference shielding coatings, flexible sensing interfaces, electroactive energy-related coatings, and biomedical/bioelectrode coatings, are discussed as extensions of PPy’s conductivity, reversible redox activity, doping/dedoping behavior, and structural tunability. Accordingly, this review first introduces the fundamental characteristics of PPy relevant to coating design, followed by major fabrication strategies, including electrochemical deposition, chemical oxidative polymerization, solution-based processing, and hybrid composite engineering. The subsequent sections analyze surface-protection mechanisms and representative emerging coating functions, with emphasis on the influence of morphology, dopants, interfaces, and composite architectures on coating performance. Finally, current challenges and future perspectives are summarized to guide the rational design of next-generation PPy-based functional coatings with enhanced durability, multifunctionality, and practical applicability.

## 2. Fundamentals of Polypyrrole-Based Functional Coatings

### 2.1. Structure and Conductivity of Polypyrrole

**Figure 1:**
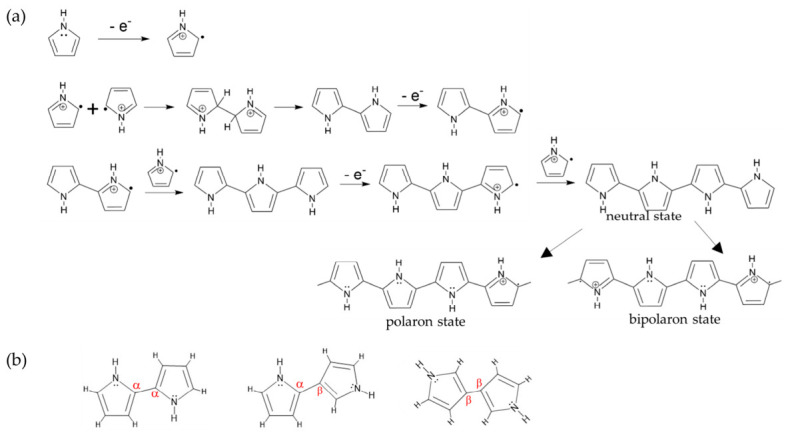
(**a**) The electrochemical polymerization mechanism of pyrrole and the structural evolution of the polypyrrole backbone among neutral, polaron, and bipolaron states upon oxidation. (**b**) Different linkage modes between pyrrole units, illustrating the ideal α–α coupling alongside α–β and β–β defect structures [18].

Polypyrrole (PPy) is one of the earliest systematically studied intrinsically conducting polymers, and its coating performance is closely related to its π-conjugated backbone, oxidative doping state, and ion-coupled redox behavior. As illustrated in Figure 1a, the formation of PPy begins with the oxidation of pyrrole monomers into radical cations, followed by coupling and deprotonation to form oligomers and eventually extended polymer chains [18]. Its main chain is typically formed through α–α coupling of pyrrole units, while α–β and β–β couplings may also occur and introduce structural defects that disrupt ideal chain regularity (Figure 1b) [18]. For coating applications, these structural features are important not as isolated polymer-chemistry details, but because they determine the continuity of charge transport pathways, the density of defects, and the stability of electroactive coating layers.

From the perspective of electronic structure, undoped PPy behaves as an organic semiconductor with a reported bandgap of approximately 3.16 eV [19]. Upon p-type oxidative doping, localized electronic states are introduced into the bandgap, generating charge carriers such as polarons and bipolarons. Ullah et al. further demonstrated by density functional theory (DFT) that p-doping significantly decreases the bandgap and electrical resistance of PPy oligomers, with the calculated energy gaps of neutral and doped 9Py systems distributed in the range of 2.91–3.41 eV [20]. This confirms that the doping level can effectively regulate the electronic structure and conductivity of PPy. Experimental spectroscopic studies have also provided direct evidence for this redox-induced structural evolution. Kaufman et al. confirmed the gradual evolution from polarons to bipolarons during electrochemical redox processes, while in situ ESR and Raman studies further revealed the electronic-state transition and the coexistence of reduced benzenoid and oxidized quinoid features during PPy oxidation [21,22,23].

The conductivity of PPy is highly dependent on its reversible doping/dedoping mechanism. Unlike the mechanism in inorganic semiconductors where charge carrier concentration is tuned by introducing trace impurities, “doping” in conducting polymers typically refers to the introduction of counterions to maintain the electroneutrality of the system following the oxidation or reduction in the main chain. For PPy, the most typical process is oxidative p-doping: the main chain loses electrons to form positive charge centers, and anions or polyanions from the solution intercalate into the film for charge compensation. During the reductive dedoping stage, these counterions will partially deintercalate, or be accompanied by the migration of cations/solvent molecules to achieve charge balance. Therefore, the electrochemical process of PPy is essentially a strongly coupled process of electron transport and ion transport. In situ electrochemical studies of PPy in aqueous solutions by Yuan et al. [24]. demonstrated that its redox characteristics are significantly influenced by the electrolyte composition, pH value, and ion types. Research by Zhou and Heinze [25] on the electropolymerization and electrochemical behavior of PPy further indicated that PPy films prepared under different polymerization conditions exhibit significant differences in oxidation wave potentials, electrochemical windows, and overoxidation tendencies.

The type of dopant not only determines the pathway of electroneutrality compensation but also profoundly affects the microscopic morphology and final electrical conductivity of the film layer. Studies by Bay et al. [26]. showed that when different alkylbenzenesulfonates are used as dopants, the electronic conductivity of PPy exhibits significant differences: the conductivity of PPy–BBS can reach up to 83 S·cm^−1^, whereas that of PPy–OBS is only 16 S·cm^−1^. This indicates that even within the same class of organic sulfonates, differences in carbon chain length, hydrophobicity, and steric hindrance can significantly alter the main chain regularity, the degree of charge carrier delocalization, and the interchain contact state. Additionally, Li et al. [27,28] synthesized PPy nanoparticles containing self-stabilized hydroxysulfoaniline units. After simple dedoping/redoping treatments, their conductivity could be broadly tuned in the range from 10^−9^ to 1.12 S·cm^−1^. This demonstrates that the doping state not only dictates the conductive switching behavior of the material but also precisely controls its intrinsic conductivity level.

In addition to dopants, the types of oxidants and solvents, as well as post-treatment processes, also significantly affect the macroscopic conductive capability of PPy. In literature reports, the conductivity of PPy spans multiple orders of magnitude: the intrinsic or weakly doped PPy reported by Zhang et al. [29] had a conductivity of only about 0.04 S·cm^−1^ (pellet) and 0.01 S·cm^−1^ (film); conversely, Machida et al. [30] achieved PPy with a high conductivity of 190 S·cm^−1^ as early as 1989 by optimizing the chemical oxidation system. Lindenberger et al. [31] reported that polypyrrole prepared by electrochemical polymerization of α-bipyrrole exhibited a room-temperature conductivity of up to 170 S·cm^−1^ under argon, but the conductivity rapidly decreased to 60 S·cm^−1^ upon exposure to air, indicating that the highly conductive state of PPy is highly sensitive to ambient atmosphere. These data indicate that although PPy can achieve high conductivity, its highly conductive state is heavily dependent on an ideal doping ratio, high main chain ordering, and low defect density, while also exhibiting sensitivity to the ambient atmosphere.

In recent years, through morphology engineering and the optimization of dispersion systems, the processability and conductivity of PPy have been synchronously improved. Water-dispersed PPy prepared by Yang and Liu [32] reached a conductivity of 2.70 S·cm^−1^ without the addition of secondary dopants; Mao et al. [33] constructed a porous interconnected PPy film using a vapor-phase polymerization strategy, further increasing its conductivity to 94 S·cm^−1^. These results confirm that when the coating simultaneously possesses a high doping level, continuous electron pathways, and efficient ion diffusion routes, PPy can balance high conductivity with excellent film-forming processability, which is of great significance for the development of high-performance functional coatings.

Although PPy exhibits high conductivity under specific conditions, most of its macroscopic samples still display the characteristics of amorphous or low-order conductive networks. X-ray diffraction (XRD) analysis of soluble PPy by Joo et al. [34] indicated that such materials exhibit typical amorphous structures. Therefore, its charge transport mechanism differs from the free electron gas model in metals; rather, it is co-dominated by intrachain delocalized transport and interchain hopping [35]. Maddison et al. [36] measured the electrical conductivity and thermopower of PPy in the temperature range of 4–350 K, pointing out that its transport behavior in the high-temperature region conforms to the Mott variable-range hopping (VRH) model. Research by Bof Bufon and Heinzel [37] on chemically synthesized PPy thin films revealed that under low electric fields, the system undergoes a transition from the Efros–Shklovskii VRH model to the Arrhenius activated transport model at around 30 K. This implies that the conduction mechanism of PPy is multifaceted and closely related to the doping level, temperature, locally ordered structure, and applied field strength of the system.

In this context, morphology engineering plays a crucial role in enhancing the electrical properties of PPy. Hao et al. pointed out that nanostructured PPy—such as nanoparticles, nanofibers, nanotubes, and hollow structures—can effectively enhance charge transport performance by shortening ion diffusion pathways, increasing the interfacial area, and optimizing the continuity of conductive networks [9]. For instance, Tavakkol et al. fabricated electrospun PPy/poly(vinyl pyrrolidone) (PVP) nanofibers with an average diameter of ~440 nm and a crystallinity of ~25.7%, achieving a maximum conductivity of 5.22 × 10^−1^ S cm^−1^ [38].

**Figure 2:**
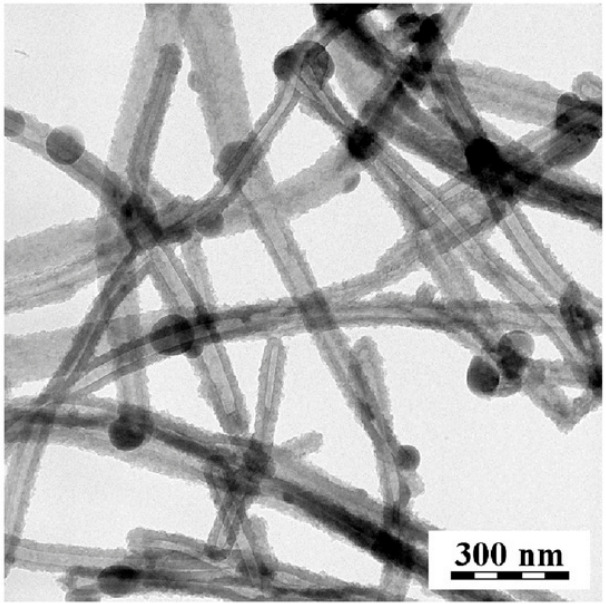
Transmission electron microscopy image of polypyrrole nanotubes, clearly showing the hollow interior of the tubular structure [45].

Focusing specifically on targeted morphological design, Czech researchers have conducted systematic and representative studies on PPy tubular structures. Kopecka et al. [39] demonstrated that under the induction of methyl orange, PPy can transition from a conventional globular morphology to nanotubes, with the resulting nanotube pellets exhibiting a maximum conductivity of 68 S cm^−1^. As shown in Figure 2, the transmission electron microscopy image clearly reveals the hollow interior of the PPy nanotubes, providing direct evidence for the formation of the tubular structure. Subsequently, Kopecky et al. [40] further optimized this synthesis system, significantly increasing the conductivity of PPy nanotubes to 91.6 S cm^−1^ and their specific surface area to 67.6 m^2^ g^−1^. Prokes et al. [41] showed that PPy nanotubes maintain reversible conductive behavior during deprotonation/reprotonation cycles; although the conductivity slightly decays after each cycle, excellent overall long-term stability is preserved.

In recent years, this strategy has been further extended to composite systems. Stejskal et al. [42] reported that pure PPy nanotubes exhibit a conductivity of ~20 S cm^−1^; upon compositing with magnetite nanoparticles, the conductivity is maintained at ~1 S cm^−1^ and can be restored to ~5 S cm^−1^ after reprotonation. Jurca et al. [43] achieved a composite conductivity of 13–25 S cm^−1^ in a MnZn ferrite/PPy core–shell system. Most recently, in 2025, Stejskal et al. [44] further highlighted that the conductivity of pure globular PPy powder under 10 MPa is merely 0.244 S cm^−1^ (and 0.288 S cm^−1^ after pelletization), whereas pure PPy nanotubes reach 14.4 S cm^−1^ and 28.4 S cm^−1^, respectively, under identical conditions. Moreover, the maximum conductivity in a tungsten/PPy nanotube composite system further escalates to 36.1 S cm^−1^.

As shown in Figure 2, the sample exhibits a distinct interwoven network of fibers or tubes, accompanied by locally distributed darker particles or nodes. This continuous, interconnected one-dimensional network facilitates the formation of more complete electron transport pathways and significantly reduces the interparticle contact resistance. Therefore, the morphological characteristics fully corroborate the conclusion that the transition of PPy from a globular to a tubular structure significantly enhances its electrical conductivity.

The charge-transport mechanism of PPy fundamentally underpins its functional responses. Because the doping/dedoping process concurrently modulates carrier concentration, counterion distribution, local conformation, optical absorption characteristics, and volumetric state, PPy exhibits quantifiable resistance or optical responses to external stimuli, including gases, humidity, strain, and applied potentials. For example, Lawaniya et al. [46] reported a flexible room-temperature ammonia sensor based on polypyrrole/functionalized multiwalled carbon nanotube (Ppy/f-MWCNT) nanocomposites. The optimized sensor exhibited a response of 26.5% toward 100 ppm NH_3_ with a response time of 16 s, and also showed high repeatability and long-term stability over the 5–200 ppm concentration range, even under extreme bending conditions. Regarding electromechanical responses, Xu et al. [47] fabricated a strain sensor based on a PPy-coated bacterial cellulose network within natural rubber. At a mere 6% PPy loading, the sensor achieved an elongation at break of 388.0% and an apparent gauge factor of 355.3 in the strain range of >300%, maintaining operational stability over 3000 cycles. Furthermore, Ratautaite et al. demonstrated that a PPy–poly(methylene blue) (PMB) electrochromic layer produced pronounced absorbance changes at 668 and 750 nm under +0.8 V/−0.8 V pulses. Subsequent optimizations of PPy-based electrochromic composite films have yielded an optical modulation of 71%, coupled with rapid coloration and bleaching switching times of 7.83 and 7.45 s, respectively.

In summary, the structural and conductive characteristics of PPy originate from its conjugated backbone, reversible doping/dedoping behavior, and the charge-transport processes dominated by polarons and bipolarons. The electronic structure of the backbone, dopant type, film morphology, and interchain packing collectively dictate its final conductivity level and modes of response to environmental stimuli. These fundamental principles not only provide the theoretical basis for understanding the surface protection, electromagnetic responses, and sensing behaviors of PPy-based functional coatings but also establish a structural foundation for subsequent performance enhancements via interfacial engineering, compositing, and morphological regulation.

From the viewpoint of coating design and performance, the electronic and redox characteristics of PPy should not be regarded as isolated material parameters. Rather, they explain how PPy participates in coating-level functions. The conjugated backbone and doped charge carriers provide continuous electron transport pathways, which are essential for antistatic coatings, electromagnetic interference shielding, electrothermal conversion, and electrical signal transduction. Meanwhile, the reversible doping/dedoping process couples electron transport with counterion migration, enabling PPy to respond to electrochemical, chemical, mechanical, and environmental stimuli. This mixed ionic/electronic transport behavior further supports redox-mediated corrosion protection, electrochromic switching, charge storage, and stimulus-responsive sensing. Therefore, PPy is more than a simple conductive additive; it can bridge passive surface protection and active functional interfaces, providing the fundamental material basis for translating molecular-level conductivity and redox activity into macroscopic coating functions.

### 2.2. Design Features of PPy-Based Coatings

In PPy-based functional coatings, actual performance is determined not only by the intrinsic conductivity or redox activity of PPy, but also by how PPy is spatially organized on or within the substrate. A highly conductive or electroactive PPy phase cannot generate stable coating functions unless it forms continuous transport pathways, maintains strong adhesion to the substrate, and retains structural integrity during service. Therefore, coating design should be regarded as the process of translating the intrinsic properties of PPy into stable macroscopic functions through morphology regulation, thickness control, surface roughness adjustment, pore-structure design, and interfacial bonding. These structural factors directly govern coating continuity, defect density, charge/ion transport, active surface accessibility, mechanical robustness, and long-term durability. From this perspective, the design of PPy-based coatings has shifted from simply depositing a conductive polymer layer to constructing an integrated interfacial structure capable of simultaneously supporting conductivity, protection, flexibility, and environmental responsiveness. Accordingly, recent studies increasingly emphasize structural configuration, film thickness and roughness, pore-structure hierarchy, and interfacial bonding states as key design parameters for PPy-based coatings [48,49,50,51,52,53,54,55,56,57,58,59,60,61,62,63,64]. In this context, PPy-based coating design has moved beyond the simple pursuit of high electrical conductivity and now focuses more on the coupling among interfacial structure, surface morphology, and multifunctional output.

**Figure 3:**
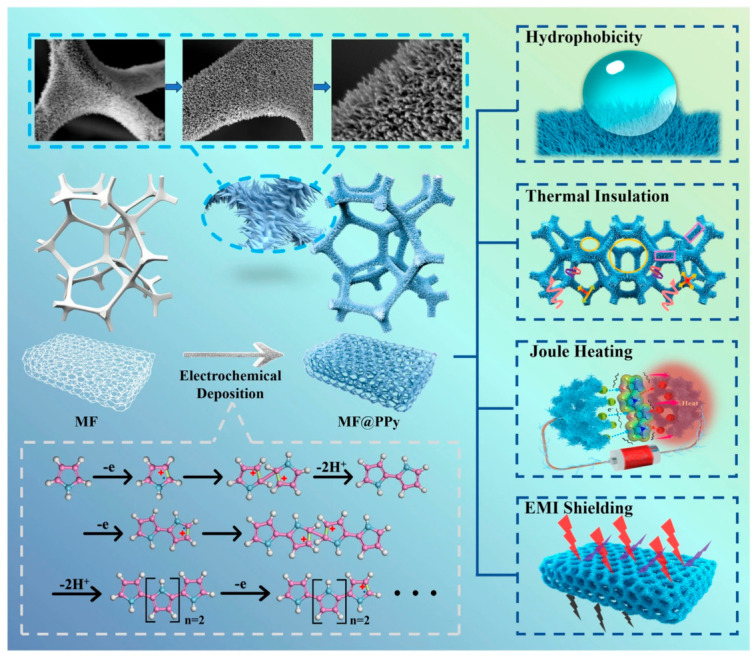
Schematic illustration of the polymerization mechanism, hierarchical structure, and multifunctional integration of the MF@PPy foam. This system constructs a 3D PPy microskeleton on the surface of a melamine foam skeleton and further grows a 1D PPy nanowire array, thereby realizing functions such as hydrophobicity, thermal insulation, Joule heating, and electromagnetic interference shielding. Reproduced/adapted from Ref [52]. Arrows indicate the polymerization/growth sequence and the transfer from hierarchical structure to functional outputs; red “+” signs denote positive charge centers generated during oxidative polymerization/doping; different schematic shapes represent the MF skeleton, PPy microskeleton/nanowire array, and the corresponding functional modules.

First, regarding structural configuration, PPy-based coatings have gradually evolved from conventional dense monolayers into complex architectures, such as core–shell, skeleton-assisted, and hierarchical porous structures. Xiong et al. [48]. uniformly introduced polydopamine (PDA) and PPy onto the surface of poly(L-lactic acid) (PLLA) nanofibers via electrospinning combined with in situ polymerization, constructing PPy/PDA/PLLA three-layer core–shell nanofibers. In this system, the shell PPy primarily imparts conductivity and photothermal conversion capabilities, while the intermediate PDA layer significantly enhances interfacial adhesion and bioactivity. Al-Shamery et al. further incorporated PPy into a nanocellulose hydrogel network to construct a flexible, interconnected 3D conductive framework, enabling environmental sensing across a broad pH range of 4–10 and demonstrating rapid responses to metal ions such as Zn^2+^, Cu^2+^, and Fe^3+^ [49]. A dynamic PPy core–shell chemomechanical actuator proposed by Yuan et al. further proved that the shell-confined PPy serves not only as a conductive outer layer but also couples electrochemical reactions with volumetric changes during redox processes to achieve actuation functions [50]. Furthermore, Wang et al. prepared flexible porous PPy films via an improved vapor phase polymerization method, demonstrating that an open pore structure is advantageous for balancing electron transport efficiency with ion accessibility [51]. A highly representative example is the hierarchical porous MF@PPy foam system reported by Liu et al.: As shown in Figure 3, this structure features a 3D PPy microskeleton constructed in situ on a melamine foam (MF) skeleton, upon which a 1D PPy nanowire array was further grown, forming a hierarchical “micro-skeleton + nanowire array” composite structure. This system achieved a water contact angle of 142.00°, an electrical conductivity of 128.2 S m^−1^, and an electromagnetic interference shielding effectiveness (EMI SE) of 55.77 dB. These results fully demonstrate that the hierarchical configuration not only improves coating continuity and interfacial roughness but also provides essential structural support for the integration of multiple functions, including hydrophobicity, thermal insulation, Joule heating, and EMI shielding [52]. Similarly, Jiao et al. obtained a flexible composite structure by in situ polymerizing PPy on pre-oxidized polyacrylonitrile (PAN) felt. The resulting material exhibited a resistivity of 24.55 kOhm cm and a pressure sensitivity of 14.90 kPa^−1^, indicating that fiber skeleton assistance is a crucial strategy for unifying continuous film formation with flexible sensing performance [53].

Second, the thickness, roughness, and adhesion of PPy coatings are core design parameters that determine their practical viability. For flexible substrates, an excessively thick PPy layer is prone to embrittlement and delamination, whereas a layer that is too thin may result in discontinuous surface coverage or localized loss of conductivity. Gleissner et al. prepared a uniform PPy coating with a thickness of approximately 0.5–1 um on polyamide fabrics and successfully applied it to wearable personal protective equipment for gas sensing, demonstrating the high feasibility of obtaining thin and continuous conductive layers on textile substrates [54]. For rigid inorganic substrates, Ismail et al. proposed a one-step aqueous electropolymerization method capable of forming a uniform, well-adhered PPy ultrathin film (approximately 9–70 nm thick) on silicon surfaces with minimal pretreatment, showcasing the enormous potential of PPy in localized surface functionalization and interfacial modification of micro/nano devices [12]. Simultaneously, the decisive role of surface roughness in the adhesion of PPy coatings has been directly verified. Studies by Oubella et al. on ABS substrates showed that the adhesion rate of the PPy coating on untreated surfaces was a mere 0%; this value increased to 66% after mechanical sanding, and reached 100% when combined with subsequent chemical etching. This indicates that a synchronized improvement in surface roughness and hydrophilicity can significantly enhance the interfacial bonding between the PPy coating and the substrate [55]. From the perspective of deposition kinetics, de Boer and Schroen utilized surface plasmon resonance (SPR) to monitor the early growth processes of PPy ultrathin films (<30 nm) in real time. They pointed out that the deposition method and dopamine copolymerization behaviors significantly influence nucleation and initial growth kinetics, thereby dictating the continuity and surface state of the ultrathin PPy film [56]. Additionally, patterning is gradually emerging as a vital direction in PPy coating design. Carcione et al. reported a one-step, mask-less PPy-polydopamine patterning strategy tailored for organic field-effect transistor platforms, indicating that PPy coatings can not only achieve large-area uniform coverage but are also advancing toward localized precise deposition and targeted device integration [57].

Third, the design of PPy-based coatings is increasingly emphasizing interfacial hybridization and multifunctional synergy—namely, achieving the comprehensive optimization of conductivity, protection, wettability control, thermal management, and antibacterial properties by introducing inorganic phases, nanoparticles, or secondary polymer components. Wang et al. constructed a photothermal superhydrophobic coating based on PPy-modified boron nitride, which exhibited an excellent photothermal effect combined with surface hydrophobicity in anti-icing/de-icing applications. This proved that PPy can act simultaneously as a functional component and an interfacial modulation agent [58]. Liu et al. reported a Au nanoparticle-PPy composite coating designed for Ti-based porous transport layer anodes. By focusing on enhancing conductivity and corrosion resistance, this work exemplified the synergistic interfacial design rationale between noble metal nanophases and PPy [59]. In the field of bipolar plates for fuel cells, Narasimharaju et al. investigated the application of TiN/CrN nanoparticle-doped PPy coatings on 5052 AA and other aluminum alloy bipolar plates. Their results demonstrated that such hybrid coatings significantly increase the corrosion resistance, polarization resistance, and overall protective efficiency of the substrate while concurrently improving electrical conductivity [60,61]. Padmanaban et al. applied a nanoparticle reinforcement strategy to PPy coatings on leather surfaces; the dispersion coatings containing nanoparticles achieved conductivities of 4.69 × 10^−4^ and 5.57 × 10^−4^ S cm^−1^, realizing nearly a twofold enhancement in conductivity compared to the pure polymer coating without nanoparticles, alongside superior hydrophobicity and antibacterial activity [62]. Furthermore, Sahoo et al. constructed a stearic acid-treated PPy superhydrophobic coating on the surface of an Mg-Ce alloy, yielding a water contact angle of approximately 153° and a corrosion inhibition efficiency exceeding 85%. This further corroborates that PPy-based coatings can achieve the synergistic optimization of multiple functions—such as corrosion resistance, hydrophobicity, and biocompatibility—through tailored surface chemical modifications [63].

Overall, the design philosophy for PPy-based coatings has profoundly shifted from simply “depositing a conductive film” to “constructing a multidimensional interfacial structural system”. On one hand, the structural configuration dictates whether PPy forms a dense film, a porous film, a core–shell structure, or a skeleton-assisted architecture. On the other hand, thickness, roughness, and adhesion determine whether the film layer is continuous, stable, and adaptable to practical substrates. Meanwhile, interfacial hybridization strategies dictate whether the coating can further satisfy a combination of stringent demands, including high conductivity, robust protection, flexible adaptability, and environmental responsiveness. Therefore, for the future design of PPy-based functional coatings, the true core challenge does not lie in the blind pursuit of singular breakthroughs in conductivity values. Instead, it lies in constructing comprehensive functional surface systems that are genuinely “processable, adherable, and capable of stable service” through the rigorous design of structural hierarchies, the reinforcement of interfacial bonding, and the synergistic enhancement of multiphase components.

### 2.3. Core Advantages and Application Limitations of PPy-Based Coatings

The core advantages of PPy-based coatings can be summarized in five aspects: conductivity, redox activity, mixed ionic/electronic transport, structural tunability, and interfacial adaptability. First, the conjugated backbone and doped charge carriers enable PPy to construct continuous conductive pathways, which are essential for antistatic coatings, EMI shielding, electrothermal conversion, sensing, and electroactive interfaces. Second, the reversible redox behavior of PPy allows it to participate in interfacial electrochemical reactions, making it particularly attractive for active corrosion protection and electrochemical passivation. Third, its ion-coupled doping/dedoping behavior provides a direct mechanism for converting chemical, mechanical, humidity, gas, or electrical stimuli into measurable resistance, optical, or volumetric responses. Fourth, PPy can be processed into dense films, nanoparticles, nanotubes, nanowires, porous networks, and core–shell structures, enabling coating designs with tunable roughness, porosity, and interfacial area. Finally, PPy can be deposited or polymerized on diverse substrates, including metals, polymers, textiles, and three-dimensional porous scaffolds, which gives it broad substrate adaptability compared with many conventional coating systems.

As summarized in Table 1, the distinct value of PPy-based coatings lies in their ability to integrate passive protection, active electrochemical regulation, and multifunctional interfacial response within one coating system. This integrated feature differentiates PPy from conventional protective coatings and makes it particularly suitable for next-generation functional coating applications.

Compared with other conducting polymers such as PANI and PEDOT, PPy shows both competitive advantages and intrinsic limitations in coating applications. PANI has been widely studied for anticorrosion coatings because of its redox activity, low cost, and ability to promote passive oxide formation on metal substrates. However, its conductivity and electrochemical state are strongly affected by protonation level and environmental pH, which may limit its stability under neutral or alkaline service conditions. PEDOT and other polythiophene derivatives generally exhibit excellent environmental stability, high conductivity, and good optical/electronic properties, especially in flexible electronics, transparent electrodes, and optoelectronic coatings. Nevertheless, they often require specific monomer structures, dopants, or formulation systems. In contrast, PPy can be readily prepared by electrochemical deposition or chemical oxidative polymerization under relatively mild conditions and can be directly formed on metals, polymers, textiles, and porous substrates. Therefore, PPy is particularly competitive in applications requiring in situ coating formation, active interfacial redox behavior, morphology regulation, and broad substrate adaptability. However, PPy is not universally superior to PANI or PEDOT; its brittleness, adhesion sensitivity, and possible dedoping-induced conductivity decay still need to be addressed through composite design, interface engineering, and dopant stabilization.

Based on the discussions in Section 2.1 and Section 2.2, the primary advantage of PPy-based functional coatings extends beyond providing a conductive pathway. It lies in the integration of the electron transport capacity of the conjugated backbone with the interfacial controllability afforded by multidimensional coating structures, facilitating a combined output of electrical, thermal, and mechanical responses. This structure-property coupling is highly applicable in flexible electronics and wearables. Studies by Lee et al. [64]. Muhammad et al. [65], and Ebadi et al. [66] have shown that fabricating PPy composite layers on flexible substrates—such as wool felt, polydimethylsiloxane (PDMS), or cotton fabrics—enables effective electrothermal conversion and high-sensitivity strain perception, while maintaining performance stability over multiple mechanical deformations or washing cycles. These findings indicate that with appropriate interfacial design, PPy can utilize the stress-buffering and structural support of flexible substrates to translate microscopic electrical properties into stable macroscopic multifunctional outputs.

Furthermore, PPy-based coatings exhibit considerable physicochemical interfacial activity. Unlike traditional pure metals or insulating polymers, PPy not only conducts electrons but also participates in interfacial physicochemical processes due to its mixed ionic-electronic conductivity. Piccioni et al. [67] observed that PPy-coated fabrics possess antibacterial capabilities, although their conductivity fluctuates due to washing-induced dedoping, indicating a coupling between surface biochemical functions and the intrinsic conductive state. In the energy and biomedical fields, Niu et al. [68] and Sevcencu et al. [69] noted PPy’s potential in reducing the contact resistance of metal bipolar plates, enhancing interfacial corrosion protection, and serving as compliant bioelectrodes. This suggests that PPy functions as an active interfacial layer, participating in processes such as sensing, antibacterial action, corrosion inhibition, and electrical stimulation while providing a conductive network.

Despite these advantages, the practical use of PPy-based coatings is still restricted by several closely related limitations. The rigid π-conjugated backbone of PPy often leads to brittleness and limited deformation tolerance, which may cause microcracking or loss of coating continuity under bending, friction, washing, or long-term service. In addition, the adhesion of PPy to different substrates is highly dependent on surface chemistry, roughness, pretreatment, deposition route, and coating thickness; insufficient interfacial bonding can result in delamination and provide preferential pathways for water, oxygen, and aggressive ions. Moreover, the conductivity and redox activity of PPy are strongly associated with its doping state, making the coating susceptible to dedoping, overoxidation, dopant migration, and conductivity decay during prolonged immersion, electrochemical cycling, or harsh environmental exposure. Therefore, improving mechanical robustness, interfacial adhesion, and long-term redox/conductive stability is essential for translating the intrinsic advantages of PPy into reliable coating performance. These limitations are summarized in Table 2.

These limitations have been observed in different PPy-based coating systems. The relatively high rigidity of the main chain (as discussed in Section 2.1) leads to macroscopic intrinsic brittleness and a tendency for microcracking. Concurrently, the doping mechanism that provides its responsiveness makes it susceptible to conductivity decay caused by dedoping in aqueous environments [69]. For example, while the PPy@PU electronic skin developed by Fu et al. [70] integrated multiple responsive functions, its long-term service stability and load-bearing capacity depended primarily on the elastic support of the polyurethane substrate. Mametja et al. [71] also indicated that PPy is rarely used as a pure monolithic film to independently withstand complex service conditions. Therefore, the independent mechanical strength and environmental stability of PPy coatings are limited and typically require compensation through 3D skeleton assistance, elastomer compositing, or nanophase hybridization (as outlined in Section 2.2).

From an engineering perspective, these limitations introduce challenges for scalable manufacturing. Reviews by Dallaev [72] and Meenakshy et al. [73] indicate that batch-to-batch variations in film quality, the long-term stability of dispersion systems, and the uniformity of large-area films are primary issues to address for the industrial application of PPy coatings. In summary, Section 2.1 explains the material foundation for PPy’s conductivity and responsiveness, while Section 2.2 demonstrates how coating structure and interfacial design translate these intrinsic properties into practical functions. The limitations discussed in this section further clarify the focus of the subsequent Section 3: how to controllably and stably construct PPy-based composite coating systems that possess multidimensional functions and operate reliably over the long term.

## 3. Fabrication and Structural Design Strategies

### 3.1. Electrochemical Deposition

As indicated in Section 2.1 and Section 2.2, the high suitability of polypyrrole (PPy)-based functional coatings for electrochemical deposition stems from the intrinsic electrochemical characteristics of their backbone oxidation, doping, and film-forming processes. Compared to chemical oxidative polymerization, electrochemical deposition utilizes an applied potential or current to directly induce the oxidation of pyrrole monomers on a conductive substrate, achieving in situ nucleation, growth, and doping. This enables precise control over film thickness, deposition rate, oxidation state, and spatial localization of the coating. Recently, this method has advanced from conventional potentiostatic and galvanostatic modes to pulsed deposition and in situ kinetic monitoring. For example, de Boer et al. investigated the early-stage growth of ultrathin PPy films (<30 nm) in real-time using surface plasmon resonance. By comparing galvanostatic, potentiostatic, pulsed galvanostatic, and pulsed potentiostatic modes, they revealed that the formation and polymerization rate of pyrrole radicals accelerated significantly only when a threshold potential of approximately ±0.4 V was reached. Furthermore, although dopamine copolymerization facilitated initial radical formation, it shifted the polymerization site from the substrate surface to the solution phase, thereby reducing the surface film-forming rate [56]. Similarly, Ismail et al. employed a dual pretreatment strategy combining plasma and heating to directly electrodeposit uniform and well-adhered PPy ultrathin films (9–70 nm) onto silicon substrates with a native oxide layer. This demonstrated that electrochemical deposition is applicable not only to metallic substrates but also to the localized functionalization of semiconductor surfaces [12].

For the construction of protective functional coatings, the core advantage of electrochemical deposition lies in its ability to drive the in situ growth of PPy directly on the target metal surface, simultaneously achieving film formation, doping, and interfacial protection. Ben Jadi et al. electropolymerized sodium saccharin-doped PPy coatings on AISI 316L stainless steel bipolar plates using a current density of 2 mA·cm^−2^ for 5, 10, and 30 min. The results indicated that the 30 min sample exhibited optimal corrosion resistance, along with the formation of a denser “cauliflower-like” surface microstructure [74]. For aluminum alloy substrates, Lin et al. evaluated the electrodeposition behavior of sulfuric acid, p-toluenesulfonic acid (pTSA), and 2-naphthalenesulfonic acid (2NS) doping systems on AA2024-T3. The PPy coatings formed in 0.2 M pTSA and 0.1 M 2NS exhibited significantly better corrosion resistance than the bare substrate. When combined with a topcoat, the overall protective efficiency of the 2NS-doped PPy reached 99.99% [75]. Furthermore, Jiang et al. fabricated a PPY/ZIF-67 composite electrodeposited coating on 430 ferritic stainless steel. The coating prepared with a 0.4 mol·L^−1^ pyrrole concentration achieved a corrosion inhibition efficiency of 99% in 0.1 mol·L^−1^ HCl and maintained durable protection for 744 h. This indicates that electrochemical deposition is particularly well-suited for constructing hierarchical composite protective interfaces consisting of an “inner conductive polymer + outer active nanophase” [76]. In the field of conductive protective layers, Gong et al. introduced graphene quantum dots into a graphene/PPy electrodeposition system to promote the uniform co-deposition of graphene. The optimized composite coating achieved an electrical conductivity of 175 S·cm^−1^, a reduced interfacial contact resistance of 8.37 mΩ·cm^2^, and a corrosion current density of 0.84 μA·cm^−2^. This confirms the distinct advantages of electrochemical deposition in synergistically optimizing high conductivity, low contact resistance, and anti-corrosion performance [77].

**Figure 4:**
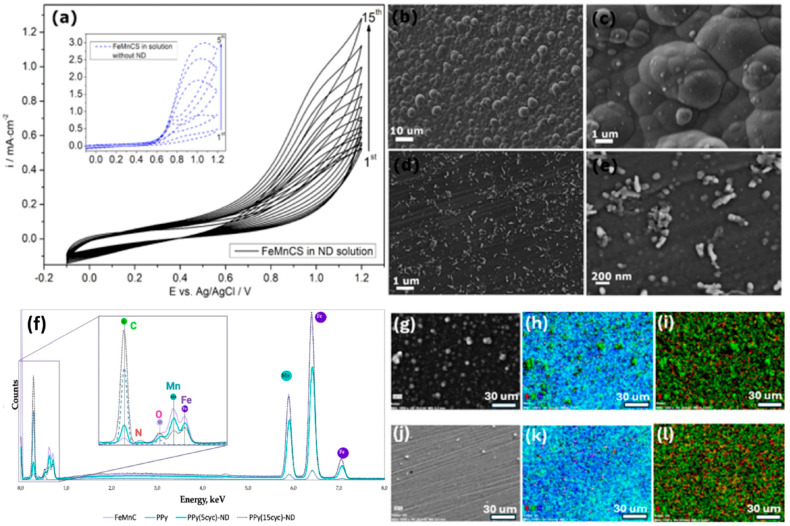
Electrodeposition and characterization of PPy-nanodiamond (ND) composite coatings on FeMnC alloys. (**a**) Cyclic voltammograms (CV) showing the stable in situ growth of the polymer film over 15 scanning cycles. (**b**–**e**) SEM images revealing the classical dense, cauliflower-like morphology of the PPy matrix and the embedded ND particles. (**f**–**l**) EDX spectrum and elemental mapping confirming the successful and uniform incorporation of NDs within the composite coating. Adapted from ref [79]. The line legend in panel (**f**) follows the original source, and the colored maps in panels (**g**–**l**) denote the spatial distributions of the corresponding elements shown in the original EDX mapping images.

Electrochemical deposition also demonstrates unique value for reactive metals and 3D porous substrates. Xu et al. prepared a PPy/CaP composite coating on a magnesium alloy using a continuous two-step cathodic deposition strategy. This method bypassed the need for pre-passivation to obtain a strongly adhered PPy coating, significantly delaying corrosion and reducing the corrosion current density while enhancing biocompatibility [78]. In a recent study focusing on highly active degradable metals, Cysewska et al. presented a novel electro-assisted method for integrating functionalized nanodiamonds (NDs) into PPy coatings on FeMnC alloy surfaces intended for cardiovascular stents (Figure 4). Utilizing sodium salicylate as a passivating agent, the in situ electropolymerization process was monitored via cyclic voltammetry (CV) (Figure 4a). The progressive increase in redox peak currents with continuous scanning visually confirmed the continuous and stable growth of the composite polymer film on the highly reactive alloy surface. Furthermore, microstructural analyses (Figure 4b–e) revealed that the coating perfectly retained the classical dense, “cauliflower-like” spherical morphology of PPy, with visible embedded ND particles. Combined with energy-dispersive X-ray (EDX) spectroscopy and elemental mapping (Figure 4f–l), it was further unequivocally confirmed that the NDs were successfully and uniformly incorporated throughout the PPy matrix. This electrodeposition-based composite interface design provides a tunable degradation barrier and establishes a versatile platform for advanced bio-interfaces on degradable implants [79]. For complex 3D structures, García-Cabezón et al. directly electrodeposited PPy and PPy-AgNPs composite coatings onto porous Ti substrates. The rough inner porous surface facilitated the deep-penetration adhesion and uniform coverage of PPy. After 90 days of immersion in PBS, hydroxyapatite successfully formed on the sample surface. Moreover, the antibacterial inhibition zone diameter increased from 5.5 ± 0.4 mm for the bare substrate to 8.2 ± 0.6 mm for the PPy coating, and further expanded to 12.5 ± 0.7 mm for the PPy-AgNPs coating [80]. In summary, electrochemical deposition is not only the most classical fabrication route for PPy coatings but also a strategy that profoundly exemplifies the unified relationship among “molecular electrochemical properties—in situ interfacial film formation—final surface functionalities”. This technique allows for precise control over the thickness and morphology of dense films and is highly adaptable for constructing composite functional interfaces on reactive metals and 3D porous substrates.

### 3.2. Chemical Polymerization and Solution Processing

Unlike electrochemical deposition, which requires conductive substrates and external electric fields, chemical oxidative polymerization utilizes oxidants (e.g., FeCl_3_, persulfates) to directly induce the polymerization of pyrrole monomers at or near the substrate interface. This approach is particularly well-suited for the comprehensive coating of insulating polymers, paper-based materials, textiles, and complex three-dimensional scaffolds. Furthermore, it exhibits high compatibility with large-area processing techniques such as dip coating, spray coating, spin coating, and printing. For instance, Flores León et al. fabricated PPy coatings on extruded polylactic acid (PLA) films via in situ chemical polymerization. A dense and uniform surface layer formed within 1 h, increasing the electrical conductivity of the PLA–PPy films to 0.022 S·cm^−1^ while simultaneously enhancing the mechanical modulus. This demonstrated that surface functionalization of polymer films can be achieved without relying on conductive substrates [81]. Mahelová et al. systematically investigated the in situ coating of polyurethane anisotropic electrospun mats, concluding that the type of oxidant and the polymerization time concurrently dictate the specific conductivity, surface free energy, thickness, and micro-morphology of the PPy layer, determining whether the coating forms a continuous layer or granular deposits [82]. Similarly, Zheng et al. initiated in situ pyrrole polymerization on cellulose nanofiber films, confirming that the “wetting-oxidation-film formation” route is highly applicable to bio-based, large-area thin-film substrates [83].

**Figure 5:**
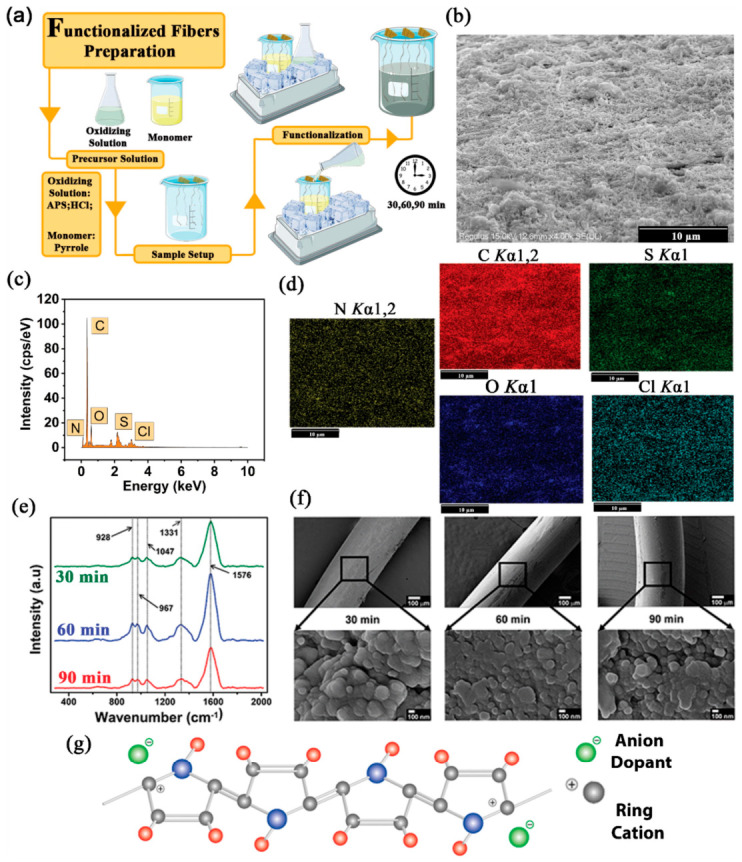
In situ chemical oxidative polymerization process and structural evolution of PPy coatings on fiber substrates. (**a**) Schematic illustration of the fiber functionalization process using the APS and HCl system. (**b**) Typical SEM image of the PPy-modified fiber surface. (**c**,**d**) EDX spectrum and corresponding elemental mapping of C, S, N, O, and Cl, confirming the uniformity of in situ doping. (**e**) Raman spectra of the coatings at different polymerization times (30, 60, and 90 min). (**f**) SEM images at various magnifications demonstrating the morphological evolution of the PPy coating from initial nucleation to dense coverage over time. (**g**) Schematic of the PPy molecular structure with anion dopants. Adapted from ref [86].

For fibrous, porous, and flexible scaffolds, the advantages of in situ polymerization are primarily reflected in comprehensive surface accessibility and synergistic morphological control. Chai et al. in situ polymerized PPy onto cellulose nanofibers (CNFs) followed by vacuum filtration to prepare CNF–PPy films. The resulting films demonstrated excellent dispersion and storage stability in aqueous media, exhibiting a conductivity of 3.91 S·cm^−1^ and an average electromagnetic interference (EMI) shielding effectiveness of 28.1 dB in the X-band. Following the incorporation of MXene, the shielding effectiveness significantly improved to 45.8 dB [84]. Poursharifi et al. chemically polymerized PPy on PA6 nanofibers and found that 1 h of polymerization with NaPTS and LiClO_4_ dopants yielded conductivities of 1.76 ± 0.12 and 4.47 ± 0.31 S·cm^−1^, respectively. Extending the polymerization time to 24 h further enhanced the conductivity by over 90% [85]. Furthermore, to elucidate the dynamic evolution of coating growth during in situ polymerization, Ferreira et al. [86] detailed the complete chemical oxidative polymerization process on fiber substrates using an ammonium persulfate (APS) and hydrochloric acid (HCl) system (Figure 5). As illustrated in the preparation schematic (Figure 5a), the process is conducted in an ice bath, where precise control over the polymerization time (30, 60, and 90 min) enables efficient regulation of surface functionalization. Microstructural observations (Figure 5b,f) clearly demonstrate that as the polymerization time increases, the PPy coating progressively evolves from initial island-like nucleation to a dense, continuous nodular covering. Concurrently, energy-dispersive X-ray (EDX) spectroscopy and elemental mapping (Figure 5c,d) confirm the uniform distribution of carbon, nitrogen, oxygen, and dopant-derived sulfur and chlorine throughout the coating. Supported by the stable evolution of characteristic peaks in the Raman spectra (Figure 5e) and the theoretical molecular structure model (Figure 5g), this work visually corroborates that chemical oxidative polymerization can construct structurally intact and uniformly doped conductive polymer networks on complex fiber surfaces through a straightforward “wetting-oxidation-time control” strategy. In continuous textile processing scenarios, Zhang et al. utilized polyester braided cords as substrates and achieved a conductivity of 63 S·m^−1^ through orthogonal optimization, maintaining robust conductive stability under low-load friction, stretching, and bending conditions [87].

From the perspective of industrial scale-up and formulation, aqueous emulsions and conductive ink systems have advanced PPy toward storable, coatable, and printable processing paradigms. Boštíková et al. coupled acrylate emulsion polymerization with pyrrole oxidative polymerization to develop a one-pot synthesis of polyacrylate latex containing a PPy component. The formulation with 0.25 wt.% pyrrole exhibited an optimal balance among emulsion stability, chemical resistance, and protective performance, maintaining structural integrity after 72 h of immersion in pH 2–8 buffers [88]. Yin et al. developed a VOC-free aqueous composite latex coating using PPy as the conductive phase and carboxylated styrene-butadiene rubber (XSBR) as the flexible binder. The resulting films achieved a conductivity of approximately 2 × 10^−3^ S·cm^−1^ and could be directly applied onto plastics, glass, rubber, and curved substrates, highlighting the distinct advantages of aqueous systems for coating complex geometries [89]. Regarding conductive inks, Mutharani et al. synthesized a green SCS:PPy conductive ink by replacing conventional PSS with sulfated chitosan. Screen-printed carbon electrodes modified with this ink achieved a low limit of detection of 1.02 nM for carbendazim [90]. Shetty et al. formulated a water-based, screen-printable ink by combining PPy with PEDOT:PSS and rGO. Micro-supercapacitors fabricated on PET exhibited areal capacitances up to 219.7 mF·cm^−2^, and humidity sensors constructed from the same ink system demonstrated rapid response times, indicating a successful transition of PPy systems toward device-friendly formulations [91].

Among typical solution-based methods, spin coating, dip coating, and spray coating address varying areal scales and device requirements. Sonker et al. prepared PPy thin films on glass substrates via spin coating for room-temperature NO_2_ sensing. The study noted that the response intensity was highly dependent on ambient humidity, indicating that while spin coating rapidly yields uniform films, the gas-sensing performance remains constrained by the film’s microstructure and the external environment [92]. In dip-coating applications, Xia et al. dip-coated an in situ polymerized MXene/PPy composite layer onto a PDMS sponge skeleton. The resulting piezoresistive sensor demonstrated a high sensitivity of 6.8925 kPa^−1^ in the 0–15 kPa range and remained stable after 5000 cycles, verifying that dip coating is exceptionally suitable for the bulk modification and construction of conductive networks in 3D porous substrates [93]. For flexible fabrics, Wei et al. employed a two-step “in situ growth + PDMS coating” approach to engineer multifunctional interfaces on silk fabrics, integrating conductivity, hydrophobicity, photothermal properties, and antibacterial activity. Aided by near-infrared irradiation, the samples achieved antibacterial rates exceeding 99.9% against both *E. coli* and *S. aureus* [94]. In spray-coating processes, Shen et al. sequentially sprayed MXene and an in situ polymerized PPy layer to fabricate spandex fabric sensors. The sensors exhibited a high response of 647.86% to NH_3_ with a relative standard deviation of only 4.85%, demonstrating that the synergy of spray coating and subsequent polymerization is highly compatible with the rapid, large-area manufacturing of flexible substrates [95].

Beyond conventional wet processing, chemical polymerization demonstrates significant advantages for functionalizing industrial microvias and complex interfaces. Zhang et al. constructed a PPy conductive seed layer on the walls of printed circuit board (PCB) blind via in situ polymerization. The layer exhibited a sheet resistance as low as 1.45 kΩ/sq and enabled the dense superfilling of copper within 70 min. Thermal shock tests further confirmed the excellent adhesion of the electroplated copper [96]. Overall, the core value of chemical oxidative polymerization and solution processing lies not in achieving the high spatial controllability of electrodeposition, but in their adaptability to non-conductive substrates, complex morphologies, and large-area continuous manufacturing. From the authors’ perspective, the most important issue in this route is not simply increasing the number of applicable substrates, but balancing film continuity, dopant stability, process reproducibility, and long-term service performance. Therefore, future development should focus on optimizing oxidant/dopant systems, improving emulsion and ink stability, and integrating wet-coating techniques to enable scalable and reliable PPy-based functional coatings under practical manufacturing conditions [97,98].

### 3.3. Composite and Hybrid Coating Design

While Section 3.1 and Section 3.2 detailed the controllable film formation of PPy on various substrates via electrochemical deposition and chemical oxidative polymerization, monolithic PPy coatings still face common limitations under realistic service environments. As outlined in Section 2.3, these include inherent brittleness, substrate-dependent adhesion, dedoping-induced conductivity decay, limited long-term stability, and uncontrolled defect formation in large-area applications. Consequently, composite and hybrid coating designs have become the primary approaches to mitigate the performance shortcomings of singular PPy systems. By integrating PPy with flexible polymer matrices, two-dimensional barrier materials, inorganic nanoparticles, corrosion inhibitors, or other functional components, these hybrid systems can retain the intrinsic conductivity and redox activity of PPy while improving mechanical robustness, interfacial bonding, barrier performance, and environmental durability. Studies indicate that compositing PPy with polymer matrices [e.g., epoxy, waterborne polyurethane (WPU), polydimethylsiloxane (PDMS)], or hybridizing it with two-dimensional (2D) materials [e.g., MXene, graphene oxide (GO), g-C_3_N_4_] and inorganic phases (e.g., ZIFs, SiO_2_, ZnO, Fe_3_O_4_, nanodiamonds) can modulate the mechanical state, shielding effect, and interfacial adhesion of the system while retaining the intrinsic conductivity and redox activity of PPy. The underlying design rationale relies on the resin phase to provide continuous film formation and a stress-buffering network; 2D nanosheets to extend the diffusion pathways of corrosive media; and inorganic particles to facilitate active inhibition, interfacial polarization, or structural support. Concurrently, PPy acts synergistically to construct conductive pathways and provide electrochemical passivation.

In resin-based composite systems, PPy is typically embedded within the continuous polymer matrix as a functional filler or dispersed conductive phase. This alters its stress state compared to a standalone film, mitigating mechanical brittleness and microcrack propagation. Sun et al. [99] constructed a sandwich-structured GO/BTA/PPy functional filler. Upon adding 0.15 wt% of this filler to an epoxy-modified polyurethane, the system demonstrated a synergistic effect of physical shielding and chemical inhibition. Similarly, He et al. [100] dispersed PPy@ZIF-8 nanoparticles into an epoxy resin. The |Z|_0.01_ Hz of the coating maintained at 3.7 × 10^8^ Ω·cm^2^ after 30 days of immersion, indicating that encapsulating the metal–organic framework (MOF) with a PPy shell helps preserve the controlled release function of the nanocontainers and the passivated state of the matrix. Gou et al. [101] utilized mesoporous PPy-coated ultrathin g-C_3_N_4_ to construct 2D nanocontainers; the low-frequency impedance of the resulting waterborne epoxy coating in the late stage of immersion was approximately three orders of magnitude higher than that of the control group. The same team later co-introduced PPy nanotubes/BTA@polydopamine and g-C_3_N_4_ into waterborne epoxy, where the composite system exhibited self-healing characteristics during a 40-day immersion test [102]. These results reflect that the flexible support provided by the resin phase is fundamental for overcoming the intrinsic brittleness of PPy and extending the effective service life of the coating.

The introduction of 2D materials for hybridization with PPy is primarily employed to compensate for the limited shielding capability and the tendency for dedoping inherent in pure PPy films. Li et al. [103] engineered a core–shell SiO_2_@f-PPy/MXene hybrid filler and incorporated it into an epoxy resin to prepare an anticorrosive and antistatic composite coating on magnesium alloys. After 14 days of immersion, the |Z|_0.01_ Hz was maintained at 9.73 × 10^10^ Ω·cm^2^, and the surface resistivity decreased to 8.68 × 10^7^ Ω·m. Shi et al. [104] utilized in situ polymerization to intercalate PPy between Ti_3_C_2_ MXene layers, which mitigated the interlayer stacking and oxidation of MXene. The composite material achieved a minimum reflection loss of −35.5 dB at a thickness of 7.50 mm. A coating containing 0.5 wt% of this filler exhibited an |Z|_0.01_ Hz of 5.12 × 10^4^ Ω·cm^2^ after 30 days of immersion. Zhang et al. [105] dispersed a ZIF-67/GO hybrid filler in a carbon nanotube-reinforced waterborne acrylic epoxy primer, with a PPy film deposited on the outer layer to construct a ZG/DCE/P bilayer structure. The ZIF-67/GO enhanced the cross-linking density and media diffusion resistance of the primer, while the outer PPy layer modulated the release rate of the corrosion inhibitors. Xu et al. [106] integrated ZIF-8, GO, and 1,10-phenanthroline into a waterborne primer via interfacial engineering, followed by a PPy outer layer. The resulting bilayer coating shifted the corrosion potential positively by 0.36 V and 0.30 V in acidic and saline media, respectively. This demonstrates that coupling the physical barrier effect of 2D nanosheets with the anodic protection characteristics of PPy is a viable strategy for enhancing the electrochemical stability of the coatings.

Incorporating inorganic nanoparticles and constructing multiscale structures serves as an alternative approach to balance the conductivity, corrosion resistance, and mechanical strength of the coatings. Ding et al. [107] fabricated a GO/CNTs/PPy composite coating on 304 stainless steel bipolar plates. Under optimized conditions, the polarization current density of the system was 8.62 μA·cm^−2^ and the interfacial contact resistance was 130.5 mΩ·cm^2^, demonstrating the regulatory effect of 1D/2D carbon phase synergy on the conductive and anticorrosive networks. For an epoxy matrix, a Fe_3_O_4_@CNF/PPy system featuring a “magnetic loss core—1D skeleton—conductive shell” structure achieved a minimum reflection loss of −23.51 dB at a 4 wt% filler content. At 5 wt%, the tensile strength of the composite reached 89.49 MPa, a 9.7% increase over the pure epoxy resin [108]. El-Sabban and Deyab [109] investigated the role of ternary ZnO-wrapped PPy-NTs/g-C_3_N_4_ composite pigments in epoxy coatings, indicating that the introduction of ZnO enhanced interfacial polarization and that the anticorrosion efficacy followed a pattern where the ZnO-containing ternary composite outperformed the binary and pure PPy systems.

**Figure 6:**
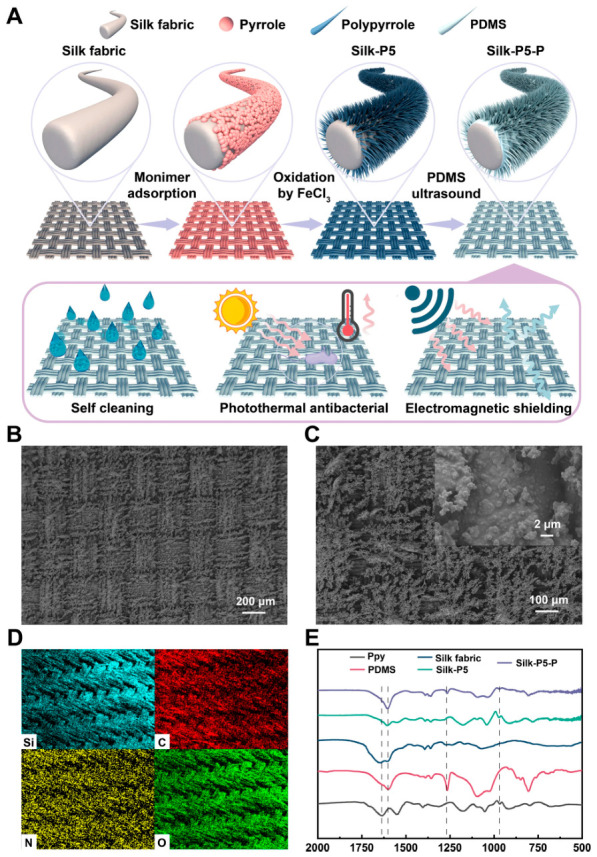
Design strategy and microstructural characterization of the multifunctional PPy-PDMS composite coating on natural silk fabrics. (**A**) Schematic illustration of the fabrication process of Silk-Ppy-PDMS composite fabrics via monomer adsorption, in situ FeCl_3_ oxidation, and ultrasonic PDMS coating, highlighting its self-cleaning, photothermal antibacterial, and electromagnetic shielding functionalities. (**B**,**C**) Typical SEM images of the optimized Silk-P5 and Silk-P5-P fabrics, revealing the one-dimensional needle-like array morphology with a high specific surface area. (**D**) EDS elemental mapping (Si, C, N, O) of the Silk-P5-P fabric surface, confirming the uniform encapsulation of the Si-containing resin over the PPy framework. (**E**) FTIR spectra evolution of the components and the composite fabrics. Adapted from ref [94]. The dotted vertical lines in panel (**E**) mark characteristic FTIR bands used to compare chemical changes among PPy, PDMS, silk fabric, Silk-P5, and Silk-P5-P.

Furthermore, coating post-treatment using polymer resins serves as an effective strategy for constructing multifunctional hybrid interfaces. To visually elucidate the design logic of this hierarchical structure, Wei et al. [94] detailed the fabrication process and microstructural characteristics of a PPy-PDMS composite coating on natural silk fabrics (Figure 6). As illustrated in Figure 6A, this hybrid system is sequentially realized through monomer adsorption, in situ oxidative polymerization by FeCl_3_, and subsequent ultrasonic coating of PDMS. Microstructural characterizations (Figure 6B,C) reveal that the in situ grown PPy exhibits a one-dimensional needle-like array, providing ample surface roughness and a charge transport network. Concurrently, energy-dispersive X-ray spectroscopy (EDS) elemental mapping (Figure 6D) and Fourier-transform infrared (FTIR) spectra (Figure 6E) confirm that the Si-containing, low-surface-energy PDMS layer is uniformly and firmly encapsulated over the PPy framework. This “conductive skeleton + flexible hydrophobic shell” hybrid architecture enables the composite fabric to achieve a superhydrophobic state with a water contact angle of 155.5°, alongside an electrical conductivity of 19.13 S·m^−1^ and an electromagnetic shielding effectiveness of 26.3 dB, while concurrently exhibiting photothermal antibacterial and self-cleaning functionalities. This demonstrates that a low-surface-energy polymer outer layer can effectively compensate for the deficiencies in the washing resistance and environmental stability of monolithic PPy while maintaining the continuity of the conductive network.

Overall, the composite and hybrid coating designs discussed in Section 3.3 are fundamentally anchored in the modulation of the physical and chemical structure of PPy itself. Regardless of the compositing materials used, the essence lies in addressing the structural limitations identified in Section 2.2 and Section 2.3 through active dopant state regulation and hierarchical interfacial engineering. First, the selection of dopants directly influences the charge carrier characteristics and environmental adaptability of PPy. For instance, the acid doping employed by Li et al. [103] in f-PPy/MXene and the small-molecule doping with p-toluenesulfonic acid validated by Wei et al. [94] both confirm the foundational role of counteranions in maintaining system conductivity. Second, interfacial transition layers facilitate compatibility among multiphase materials. Examples include Gou et al. [102] utilizing polydopamine to encapsulate PPy, and Zhang et al. [105] employing ZIF/GO hybrid structures, both of which improved filler dispersion and interfacial charge transfer within the polymer matrix. Third, hierarchical or bilayer architectures achieve the compartmentalization and synergy of functions through structural decoupling. The PPy bilayer composite coating reported by Cho et al. [110] demonstrated that employing phosphoric acid doping in the inner layer to ensure adhesion, and long-chain acid doping in the outer layer to reduce porosity, resulted in a positive shift in the corrosion potential and a reduction in the anodic current density. Therefore, the application of multiphase composite materials is not merely an additive process of preparation techniques; rather, it involves the interfacial coupling and structural reorganization of functional units across different scales, thereby integrating mechanical stability, service durability, and interfacial adhesion—properties challenging for pure PPy to possess simultaneously—into a unified protective coating system. Therefore, the key message of composite and hybrid PPy coating design is not the simple accumulation of functional fillers, but the targeted compensation of the intrinsic weaknesses of PPy through mechanical buffering, interfacial reinforcement, dopant stabilization, and hierarchical structural regulation.

## 4. PPy-Based Coatings for Surface Protection

Surface protection is a representative field in which the coating-specific advantages of PPy can be directly reflected. In practical service environments, protective coatings are required not only to block the penetration of water, oxygen, and aggressive ions, but also to maintain stable adhesion and interfacial integrity during long-term exposure. PPy-based coatings are well suited to this requirement because their protective effect arises from the coupling of barrier reinforcement, redox activity, and structural regulation. Dense PPy layers or PPy-containing hybrid coatings can extend the diffusion pathways of corrosive species, while doped PPy may participate in interfacial electron-transfer processes and facilitate the formation of passive oxide or hydroxide layers on metal substrates. In addition, the incorporation of polymer matrices, two-dimensional nanosheets, corrosion inhibitors, or inorganic particles further improves coating compactness, adhesion, mechanical robustness, and durability. Therefore, corrosion protection represents a primary example of PPy-based surface protection, while mechanical durability, wear resistance, and environmental barrier performance can be regarded as related extensions of the same structure-regulated protection strategy.

### 4.1. Anti-Corrosion Mechanisms and Performance

The anticorrosion behavior of polypyrrole (PPy)-based coatings relies not solely on the physical barrier provided by traditional organic layers against corrosive media, but rather exhibits a synergistic mechanism of physical shielding and redox passivation. On one hand, dense PPy layers or PPy-containing composite films prolong the diffusion pathways of H_2_O, O_2_, and Cl^−^ toward the metal/electrolyte interface, thereby reducing local charge transfer rates. On the other hand, doped PPy possesses reversible redox activity, which facilitates the formation of more stable oxide/hydroxide passive layers on the substrate surface through anodic protection or electron transfer processes, thereby inhibiting anodic metal dissolution. Recent studies indicate that optimizing PPy anticorrosion systems hinges on tuning dopant anions, constructing interfacial layers, and designing multiphase composites to concurrently enhance film density, passivation capability, and long-term service stability.

In single PPy layers or PPy-dominated surface layers, dopants exhibit the most direct influence on the anticorrosion mechanism. Xiao [111] prepared oxalate-doped PPy on Q235 steel surfaces via a potentiostatic method. The results indicated that effective protective films could be obtained over a broad range of deposition potentials, demonstrating that even relatively simple electrodeposited PPy can simultaneously provide interfacial shielding and anodic passivation, provided it forms a continuous and dense film. Huang et al. [112] in situ electrochemically constructed dense PPy layers with controllable thicknesses on AZ31 magnesium alloys. They noted that PPy films prepared by cyclic voltammetry (PPy-CV) were thicker and denser, consequently exhibiting superior corrosion resistance. For highly reactive degradable magnesium alloys, the PPy/Zn bilayer coating constructed by Luo et al. [113] elucidated a clear synergistic logic: the underlying Zn layer provided additional sacrificial protection, while the outer PPy layer blocked corrosive media and retarded electrochemical attack. Consequently, the bilayer system demonstrated significantly better long-term protection in Hank’s solution compared to the monolayer system. Subsequently, Luo et al. [114] optimized the outer PPy layer via salicylate (SA)/tartrate co-doping. At a NaSA concentration of 1.0 mM, the protection time reached 54 ± 1.5 days. However, excessive doping led to overoxidation and an increase in defects, reducing the protection time to 40 ± 1.0 days. This indicates that the dopant concentration must be balanced among conductivity, density, and structural stability. Furthermore, the same group [115] introduced Ag nanoparticles (NPs) into this bilayer system. The resulting Ag NPs-PPy(TA + SA)/Zn coating extended the protection time to 113 ± 2.6 days, markedly longer than the 54 ± 1.5 days observed without Ag, indicating that the nano-metallic phase effectively delays localized failure and enhances long-term service life.

Building upon this, doping with corrosion-inhibiting anions further amplifies the redox passivation characteristics of PPy. Abdoune et al. [116] compared the protective behavior of undoped PPy and MoO_4_^2−^-doped PPy on iron surfaces. They observed that the protection time for the MoO_4_^2−^-doped PPy increased to 84 h in 3% NaCl, compared to only 42 h for the undoped PPy. This result suggests that molybdate not only participates in the structural regulation of the film but also provides repassivation capabilities in localized failure areas, thereby extending the protection lifespan. Similarly, Dua et al. [117] developed a long-chain alkylphenol-functionalized PPy derived from cashew nut shell liquid for acid corrosion inhibition in 15% HCl. The incorporated pentadecyl units conferred enhanced hydrophobicity to the material and improved its inhibitory efficacy against mild steel corrosion through an interpenetrating layered coverage. These studies indicate that the anticorrosive capacity of PPy does not stem solely from its conjugated backbone; doped anions or hydrophobic side chains can significantly enhance the protective effect by improving film density, stability, and interfacial coverage.

When PPy is incorporated into organic coatings or resin matrices, the protective mechanism typically transitions from “single conductive film protection” to a dual synergy of “resin shielding + PPy passivation”. Meng et al. [118] introduced p-toluenesulfonate (PTSA)-doped PPy into an epoxy system at loadings of 4, 6, and 8 wt%. The composite coating with 6 wt% PPy (PPy-PTSA/EC-6%) exhibited optimal performance. The study indicated that PPy-PTSA promoted the formation of a dense and thicker oxide film at the aluminum alloy/coating interface, while the epoxy layer acted as a barrier against corrosive media. This established a synergy of “active passivation + passive barrier”. Ren et al. [119] proposed a polyoxometalate (Mo_7_O_24_^6−^, POM) and PPy synergistic strategy. Leveraging the dual electrochemical activities of PPy and POM, the continuous growth of a dense oxide layer on the aluminum substrate was promoted. After 150 days of immersion in 3.5 wt% NaCl, the low-frequency impedance of the PPy-POM/EC coating was maintained at 1.7 × 10^10^ Ω·cm^2^. This finding illustrates that the role of PPy in composite resin systems extends beyond merely providing conductivity; it serves as an active phase that promotes interfacial self-passivation in prolonged synergy with the barrier effect of the resin.

Compared to traditional resin composites, the hybridization of 2D materials with PPy advances the anticorrosion mechanism to a multi-coupled level involving “physical shielding + passivation + complexation/release”. Li et al. [120] synthesized phytic acid (Ph)-doped PPy/GO nanofillers via a one-pot method and introduced them into an epoxy matrix. The results showed that the GO-PPy@Ph nanoparticles dispersed uniformly within the coating, enhancing resistance to penetrant diffusion and significantly improving adhesion and mechanical properties. More importantly, after 60 days of immersion in 3.5 wt% NaCl, the |Z|_0.01_ Hz remained at approximately 10^10^ Ω·cm^2^. This excellent performance was attributed to the physical shielding of the impermeable GO nanosheets, the passivation effect of PPy, and the complexation of metal ions by phytic acid. The PPy/GO/Mo/Sal system reported by Hùng et al. [121] reflected a similar rationale: co-doping with GO, molybdate, and salicylate further reinforced the anticorrosion behavior on low-carbon steel. This indicates that incorporating 2D nanosheets alongside inhibitory anions into the PPy network is an effective pathway for enhancing the long-term protection of low-carbon steel.

The role of PPy in self-healing anticorrosion systems has also been demonstrated. Chen et al. [122] designed a cross-linked conductive PPy hydrogel network and introduced it into an epoxy system for active corrosion control on magnesium alloys. The encapsulated phytic acid inhibitor exhibited distinct pH-responsive release characteristics. At a 5 wt% loading, the coating resistance and charge transfer resistance of the epoxy coating after 28 days of immersion were maintained at 4.39 × 10^5^ Ω·cm^2^ and 8.28 × 10^5^ Ω·cm^2^, respectively. This enhancement originated from the barrier effect of the bulk epoxy, the continuous anodic protection by PPy, and the responsive release of phytic acid at crack or corroded sites. Ramezanpour et al. [123] further constructed a GO/PPy/ZIF-8 ternary hybrid architecture, where GO provided 2D shielding, PPy supplied conductive anticorrosion and interfacial passivation, and ZIF-8 managed the controlled release and self-healing functions. The system exhibited exceptional stability during long-term immersion: after 1080 days, the shielding efficiency reached 98.9% with a |Z|_0.01_ Hz of 13.8 GΩ. The self-healing efficiency reached 58.6% 24 h after scratching, alongside an 11% increase in adhesion and a 90% reduction in the cathodic delamination radius. These results verify that when hybridized with nanocontainers and 2D materials, PPy acts as a pivotal functional phase bridging physical shielding, electrochemical passivation, and active self-healing mechanisms.

For specialized service scenarios, the integration of PPy with layered inorganic phases or in bilayer functional structures also demonstrates distinct advantages. Gu et al. [124] fabricated an MMT/PPy-PCL composite coating on AZ31B medical magnesium alloy. The system achieved a protection efficiency exceeding 95% while maintaining cell viability above 90%, demonstrating that combining PPy with montmorillonite (MMT) and polycaprolactone (PCL) successfully balances anticorrosion, biocompatibility, and controllable degradation. Bian et al. [125] constructed a PPY/Ti_3_C_2_T_x_-acrylic epoxy double-layer coating on 304SS bipolar plates. In 0.2 M HCl, its corrosion potential and corrosion current density were 38 mV and 0.00927 μA·cm^−2^, respectively, outperforming both the individual PPY layer and the Ti_3_C_2_T_x_ layer. The study noted that the inner PPY layer provided anodic protection and isolated potential galvanic corrosion following damage to the outer Ti_3_C_2_T_x_ layer, while the outer MXene-epoxy layer significantly reinforced the barrier effect. This highlights the unique value of PPy as an “intermediate regulatory layer” within multilayer protective structures.

Overall, the studies discussed in this section show that the anticorrosion performance of PPy-based coatings should be understood as a coupled result of barrier shielding, redox-mediated passivation, dopant regulation, and composite-interface stabilization. From simple PPy films on steel substrates to PPy/Zn bilayers on magnesium alloys, PPy-containing epoxy coatings, and hierarchical systems involving GO, ZIF-8, MMT, or Ti_3_C_2_T_x_, recent progress indicates a clear transition from singular conductive-film protection toward integrated protective architectures. In these systems, the long-term durability of PPy-based anticorrosion coatings depends not only on the intrinsic redox activity of PPy, but also on whether the conductive/redox-active phase can be stabilized within a dense, adherent, and defect-tolerant coating structure.

### 4.2. Mechanical Durability and Wear Resistance

Distinct from the focus on corrosive media permeation and interfacial electrochemical reactions discussed in Section 4.1, the critical challenges of polypyrrole (PPy)-based coatings in mechanical protection primarily involve brittle cracking, interfacial delamination, frictional wear, conductivity attenuation, and structural instability under cyclic bending/stretching. Because pure PPy is intrinsically rigid and lacks extensibility, it is prone to microcrack initiation under repeated deformation or external friction when used as an isolated surface layer. Consequently, the contemporary research paradigm has shifted toward a composite interfacial design structured as “carrier substrate for load-bearing + PPy functional layer for conduction/response + outer protective phase for stabilization”. In this context, PPy functions primarily as a functional mechanical protective layer. The objective is not merely to increase surface hardness, but to retard structural and functional failure induced by friction, bending, washing, and cyclic loading while preserving conductivity and other surface functionalities.

First, the synergy between substrate support and in situ PPy coating represents the most direct strategy to mitigate the inherent brittleness of PPy. Kundu et al. [126] fabricated PPy-modified multifunctional coatings on polyamide 66 (PA66) textiles via in situ polymerization. The modification increased the electrical conductivity by approximately 43 times compared to neat PA66, decreased the surface water contact angle from 124.3° to 49.9°, and reduced the peak heat release rate by 45%. This indicates that while relying on the mechanical backbone of PA66, PPy significantly enhances the comprehensive service stability of the textile through continuous surface coverage and strong interfacial adhesion. Similarly, Luo et al. [127] coated 3D spacer fabrics with PPy and determined the optimal preparation conditions to be 0.10 mol·L^−1^ pyrrole, 0.40 mol·L^−1^ FeCl_3_, and 0.40 mol·L^−1^ PTSA polymerized for 2 h. For representative samples, the warp breaking strength increased from approximately 580.74 N to 907.82 N, and the weft strength from 194.29 N to 441.23 N. The study also noted that the electrical resistance of these PPy-coated fabrics stabilized after approximately 20 days in air; however, conductivity retention decreased after prolonged washing, suggesting a need to further balance mechanical reinforcement with washing durability.

A similar rationale is applicable to weavable and stretchable fibers. Lei et al. [128] continuously fabricated PPy/waterborne polyurethane (WPU)/PET composite conductive fibers by blending pyrrole with WPU for in situ polymerization on PET fibers. After treatment in 50 g·L^−1^ NaOH for 6 h, the moisture regain of PET increased from 0.45% to 1.13%. Following 5 polymerization cycles with a WPU content of 5 wt%, the fiber resistance reached 58.41 kΩ·cm^−1^. The WPU/PPy composite layer partially compensated for the mechanical degradation caused by the alkali treatment while exhibiting excellent wear and washing resistance. For highly elastic scenarios, Pan et al. [129] constructed a braided composite yarn@polydopamine@polypyrrole (BYs–PDA–PPy) strain sensor. The results showed that the sensor possessed a large workable strain range of up to 105%, exhibited a gauge factor of 51.2 in the 0–40% strain range and 27.6 in the 40–105% strain range, and maintained long-term stability during repeated deformation. These results demonstrate that the elastic yarn architecture and PDA-mediated interface do not merely “support” the PPy layer physically; rather, they act as strain-redistribution and crack-buffering elements during deformation, thereby substantially improving the repeatable stretchability of the PPy conductive layer.

**Figure 7:**
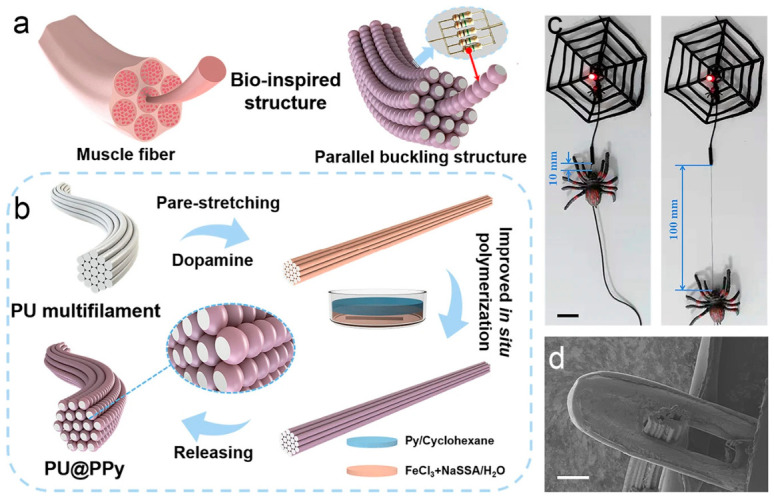
Design strategy and electrical performance characterization of the ultrastretchable conductive PU multifilament with a parallel buckled PPy structure. (**a**) Schematic illustration of the parallel conductive multifilament model inspired by skeletal muscle fiber bundles. (**b**) Schematic of the “pre-stretching—surface modification—in situ polymerization—releasing” process to fabricate monofilaments with wrinkled PPy coatings. (**c**) Optical images demonstrating the stable illumination of an LED by the composite fiber under extreme elongations from 0 mm to 100 mm (~900% strain). (**d**) SEM image showing the conductive multifilament passing through the eye of a sewing needle, illustrating its fine diameter (0.21 mm) and excellent integrability. Adapted from ref [130].

To address the issue of conductive coating fracture under large strains, Li et al. [130] designed an ultrastretchable conductive polyurethane (PU) multifilament featuring a parallel buckled PPy structure inspired by skeletal muscle fiber bundles (Figure 7). As illustrated in Figure 7a,b, this system utilized a “pre-stretching—surface modification—in situ polymerization—releasing” process to construct uniform PPy wrinkles on parallel PU monofilaments, employing sodium sulfosalicylate (NaSSA) as a plasticizer to improve the intrinsic flexibility of PPy. Microstructural analysis indicated that the parallel buckled architecture functions as a micro-resistor network during stretching; cracks initiating in the PPy coating of an individual monofilament do not propagate to adjacent ones. Macroscopic performance and morphological characterizations (Figure 7c,d) revealed that the composite fiber possessed a fine diameter of 0.21 mm and an initial conductivity of 238.0 S·m^−1^, and could stably illuminate an LED under elongations from 0 to 100 mm (~900% strain). Electrical evaluations further demonstrated a quality factor (Q) of 10.9 at 200% strain, with the relative resistance change (ΔR/R_0_) remaining at merely 3.5 even at a 900% tensile strain. This study confirms that integrating biomimetic parallel structures with interfacial pre-strain buckling engineering effectively decouples the intrinsic rigidity of conductive polymers from the tensile failure of macroscopic devices, offering a structure-oriented mechanical protection strategy for highly deformable flexible electronics.

Second, interfacial tailoring and morphological engineering are critical for enhancing the mechanical robustness and adhesion of PPy. Shukla et al. [131] developed PPy microrods on polyester fabrics via a template-free hybrid polymerization approach. Using vapor phase polymerization for just 3 min reduced the fabric resistance from approximately 200 × 10^9^ Ω to 220 Ω, forming a microrod PPy layer with a thickness of ~0.42 μm. Compared to random granular coverage, such oriented microrods or dense continuous layers are more effective at minimizing localized stress concentrations, thereby preserving structural integrity during bending and friction. Zou et al. [132] performed in situ polymerization on cotton fabrics at a low temperature of 0 °C to obtain a more uniform and compact PPy microstructure. This yielded an electromagnetic interference (EMI) shielding effectiveness of 26.4 dB—a 63.47% improvement over samples prepared at 20 °C—along with enhanced breathability, flexibility, and durability. This verifies that the mechanical service stability of PPy coatings is fundamentally dependent on the uniformity and continuity of their micromorphology and the minimization of brittle defects.

Furthermore, the introduction of an outer protective phase or a secondary functional phase advances the mechanical protection of PPy from mere crack resistance to multi-scenario durability, including resistance to wear, washing, icing, and environmental degradation. Li et al. [133] constructed a layer-by-layer PPy/CuNPs/PDMS structure on polyester nonwoven substrates. The study indicated that Cu nanoparticles (CuNPs) enhanced the continuity of the conductive pathways, while the PDMS layer provided flexible encapsulation and interfacial stabilization, significantly improving coating fastness and long-term application stability. Yuan et al. [134] introduced ZIF-8/PPy into wearable textiles, imparting superior environmental stability while maintaining breathability and flexibility. The material exhibited an icing delay time of 1291.4 s and a solar absorption rate of 90–95%. Under conditions of −20 °C and 1 sun irradiation, the surface temperature rapidly recovered to approximately 25 °C. These findings demonstrate that when PPy operates synergistically with porous inorganic phases or flexible resin layers, mechanical protection extends beyond resisting external forces to withstanding structural degradation induced by freezing, thermal-humidity cycling, and complex outdoor environments.

Plasma and plasma-jet-assisted deposition offer novel pathways for improving the adhesion, uniformity, and long-term wear durability of PPy. Heredia-Rivera et al. [135] utilized cold atmospheric pressure plasma to directly deposit PPy–Ag nanocomposite layers on textile substrates, optimizing the precursor mass ratio to 96% pyrrole and 4% AgNO_3_. This method achieved direct, uniform, and robust encapsulation of complex fiber surfaces without significantly compromising the flexibility and breathability of the textiles, enabling stable service for 10 days in wearable ECG monitoring. Al-Qahtani et al. [136] applied plasma-assisted treatment to fabricate an AgNPs/Ppyr/TMHDS layer on recycled nonwoven cotton, obtaining a conductivity of 0.6176 S·cm^−1^ and a water contact angle of 163.0°. Crucially, its fastness to perspiration, washing, light, and friction was rated “good to very good"; it retained strong photocatalytic and self-cleaning capabilities after 10 wash cycles. The authors noted that the incorporation of TMHDS and AgNPs/Ppyr did not significantly impair the inherent mechanical properties of the fabric. This confirms that interfacial activation combined with low-surface-energy outer encapsulation effectively mitigates the detachment and performance degradation of PPy under washing, friction, and hydrothermal conditions.

Beyond fundamental durability, another significant trend in the mechanical protection of PPy coatings is their deep coupling with the cyclic stability of wearable devices. Zhao et al. [137] reported an all-textile pressure sensor utilizing a conductivity-modulable PPy fabric as both the electrode and sensing layers, highlighting its breathability, biocompatibility, degradability, high sensitivity, and broad detection range. Such studies reflect a fundamental principle: stable sensing outputs can only be achieved if the PPy layer maintains a continuous conductive network during repeated compression, recovery, and human movement. Xu et al. [138] electrochemically polymerized a PPy coating on alginate nonwoven fabrics with the assistance of an ITO electrode, validating the positive role of bio-based fiber skeletons in facilitating continuous PPy film formation and strain buffering. Wang et al. [139] prepared a PPy-CNT/silk composite fabric that retained the natural softness of silk while achieving an electrical conductivity of 0.83 S·cm^−1^ and an areal capacitance of 212.8 mF·cm^−2^. After 5000 charge/discharge cycles, it retained 77.8% of its capacity, and managed to maintain ~92% capacity even in a bent state. This illustrates that for flexible energy storage or flexible electronics, “mechanical protection” of PPy manifests not only as the physical retention of the coating but also as the sustained stability of electrical and electrochemical functions under long-term mechanical perturbation.

Finally, recent studies on multilayer thermal management fabrics provide clear examples of the integrated “PPy mechanical protection—environmental protection—functional retention” paradigm. Tang et al. [140] in situ grew PPy on cotton fabrics, subsequently introducing silver nanowires (AgNWs) and PDMS to construct an asymmetric multilayer structure. This system achieved a maximum solar absorption of 98.6% in the visible region, a mid-infrared reflectance of 76.3%, and a Joule heating response of ~137 °C at 3 V. It preserved robust solar absorption, conductivity, hydrophobicity, and infrared reflectance after subjection to friction, bending, and washing. In this architecture, PDMS acts not only as a hydrophobic agent but, more importantly, as a flexible outer layer that prevents the detachment and fracture of PPy and AgNWs under external forces. Furthermore, hydrogen bonding between PPy and the hydroxyl groups of the cotton fibers enhances interfacial stability. Thus, PPy within multilayer structures acts no longer as an isolated coating, but as a critical “intermediate stabilizing layer” and functional conversion phase within the mechanical protection system.

In summary, the “wear and mechanical protection” discussed in Section 4.2 transcends the single-parameter enhancement characteristic of traditional hard wear-resistant coatings. Instead, it systematically enhances the structural integrity and functional endurance of materials subjected to friction, bending, stretching, compression, washing, and environmental cycling through three pathways: substrate load-bearing, interfacial reinforcement, and outer layer protection. Recent representative works indicate that pursuing higher conductivity in a standalone PPy layer is insufficient for long-term service. Effective strategies integrate PPy within fabric/fiber skeletons, elastomers, nanoparticles, or hydrophobic encapsulation layers to construct core–shell, hierarchical, or multiscale composite interfaces. The future trajectory of this field depends on further elucidating the mapping relationships among “PPy microstructure—interfacial adhesion—mechanical durability—functional attenuation”, thereby advancing PPy-based surface layers from merely “capable of working” to “durable in working”. This structure-oriented viewpoint is also consistent with broader studies on smart composites, contact fatigue, adaptive impact protection, graded microstructures, abrasive wear, anti-adhesive coated surfaces, and interfacial lubrication, which collectively indicate that mechanical durability is strongly governed by microstructural organization, interfacial stress distribution, surface damage evolution, and friction regulation [141,142,143,144,145,146].

### 4.3. Environmental Barrier and Surface Wettability

Unlike Section 4.1, which emphasizes the control of electrochemical failure at the metal/electrolyte interface, the application of polypyrrole (PPy)-based coatings in barrier and environmental protection focuses on the comprehensive defense against moisture, salinity, pollutants, oil fouling, ultraviolet (UV) irradiation, and complex hygrothermal environments. In this context, the role of PPy transcends traditional physical barriers to oxygen and ion diffusion. Instead, it is primarily manifested in regulating surface wettability and mass transfer behavior through the construction of continuous conductive/photothermal interfaces; mitigating pollutant adhesion and salt crystallization via compositing with hydrophobic layers, two-dimensional (2D) nanosheets, or photocatalytic materials; and maintaining stable service performance under prolonged solar exposure, immersion, washing, or harsh solution conditions. Consequently, the recent research paradigm for PPy-based environmental protection has shifted from “simple media isolation” to an integrated interfacial design encompassing “surface barrier—self-cleaning—anti-salt crystallization—anti-fouling”.

First, in the fabrication of flexible textiles and functional surface layers, coupling PPy with low-surface-energy outer shells or photocatalytic components significantly enhances the waterproofing, anti-fouling, self-cleaning, and environmental durability of the substrates. Meng et al. [147] constructed a PPy nanotubes (PNTs)/PDMS composite layer on cotton fabrics. The resulting material exhibited an electrical conductivity of 2715.8 S·m^−1^ and an X-band total electromagnetic interference shielding effectiveness of 28.2 dB, while retaining lightweight, breathable, and self-cleaning characteristics. The coating demonstrated robust stability following ultrasonication, cyclic bending, and washing, indicating that the synergy between PPy and PDMS not only optimizes electrical performance but also reinforces interfacial integrity under environmental exposure. Naysmith et al. [148] incorporated green-synthesized Ag nanoparticles and PPy into e-textiles, yielding a sample conductivity of 0.6176 S·cm^−1^ and a water contact angle of 163.0°. This composite fabric simultaneously possessed favorable UV protection, photocatalytic self-cleaning, and overall colorfastness, without significantly compromising the inherent mechanical properties of the textile. Meganathan et al. [149] applied a g-C_3_N_4_/PPy composite layer to cotton fabrics, achieving a photocatalytic degradation efficiency of 96.5% alongside pronounced stain removal and antibacterial capabilities. The study highlighted that the water volume required for per-unit-area stain cleaning under solar irradiation was only ~1 cm^2^·mL^−1^, signifying that PPy composite layers elevate “environmental protection” from passive defense to active surface self-purification.

Second, in solar-driven interfacial evaporation and desalination systems, PPy exhibits exceptional adaptability to high-humidity, high-salinity, and continuous water-contact environments, representing a defining direction in PPy-based environmental protection research. Maity et al. [150] fabricated a PPy photothermal layer on viscose nonwoven fabrics. At a PPy weight gain of ~22.3%, the surface temperature rose to 71 °C after 5 min of solar irradiation, and the maximum evaporation rate reached 3.343 kg·m^−2^·h^−1^. This confirms that a continuous PPy layer effectively enhances light absorption and localized interfacial heat accumulation. The PVA-PPy nanoparticle/hydrogel-fabric system designed by Han et al. [151] underscored the criticality of structural design for environmental stability: the system achieved a solar absorptance exceeding 92% and an evaporation rate of 2.03 kg·m^−2^·h^−1^ in pure water. In 3.5 wt% and 10 wt% saline solutions, the evaporation rates were maintained at 1.95 and 1.85 kg·m^−2^·h^−1^, respectively. Remarkably, after 100 h of continuous operation in 20 wt% high-salinity brine, the evaporation rate remained at 1.55 kg·m^−2^·h^−1^, demonstrating pronounced anti-salting and long-term stability. Yang et al. [152] incorporated PPy onto the surface of waste mop sponges to construct salt-resistant evaporators. Under 1 sun illumination, the evaporation rate reached 3.66 kg·m^−2^·h^−1^, and was maintained at 2.91 kg·m^−2^·h^−1^ in 20 wt% simulated seawater. Outdoor tests indicated that a 1 m^2^ device could produce ~15 kg of freshwater per day. These findings demonstrate that the PPy layer acts not only as a photothermal conversion center but also leverages its continuous surface network and porous support structure to inhibit the permanent accumulation of salt on the evaporative surface, thereby enabling long-term barrier protection in complex aqueous environments.

Focusing on anti-salt crystallization, acid/alkali resistance, and outdoor cyclic stability, recent PPy systems have evolved toward more refined hierarchical structural designs. Gui et al. [153] engineered a PPy light-trapping layer (CPF-2PPy) on surface-carbonized pulp foam. Across 10 cycle tests under 1 sun, the average evaporation rate reached 2.56 kg·m^−2^·h^−1^, illustrating that compositing PPy with hierarchical porous skeletons aids in maintaining long-term mass transfer channels. The generic PPy-based nanocomposite evaporator developed by Wang et al. [154] exhibited an evaporation efficiency of 94.7% under 1 sun and retained ~87.0% efficiency even after prolonged operation in 20% salinity, displaying excellent salt rejection and environmental adaptability. Although the Au@PPy/cotton fabric system constructed by Sun et al. [155] presented a relatively modest evaporation rate of 1.33 kg·m^−2^·h^−1^, its energy efficiency reached 93.38% alongside favorable acid and alkali resistance, demonstrating that noble metal/PPy synergy can effectively enhance interfacial stability in complex chemical media. Zhong et al. [156] fabricated a tree-ring-inspired 3D solar evaporator by rolling PPy-decorated waste cotton fabrics. The sample achieved a light absorption rate of ~99.00% and an evaporation rate of 2.36 kg·m^−2^·h^−1^ under 1 sun; this rate remained at 2.05 kg·m^−2^·h^−1^ in 20 wt% saline water, with a 1 m^2^ outdoor unit yielding ~10 kg of water daily. Xing et al. [157] deposited a PPy evaporative layer on polyester filter cotton, yielding an evaporation rate of 2.44 kg·m^−2^·h^−1^ under 1 sun and an average daily water production of 3.5–4.3 kg over 6 consecutive days in a closed outdoor system. Collectively, these studies reveal that the contribution of PPy to environmental protection lies not solely in photothermal conversion, but crucially in its synergy with porous skeletons, noble metal nanophases, and hierarchical structures, which substantially elevates the interfacial stability of the materials under high-salinity, acid/alkali, and continuous outdoor operational conditions.

**Figure 8:**
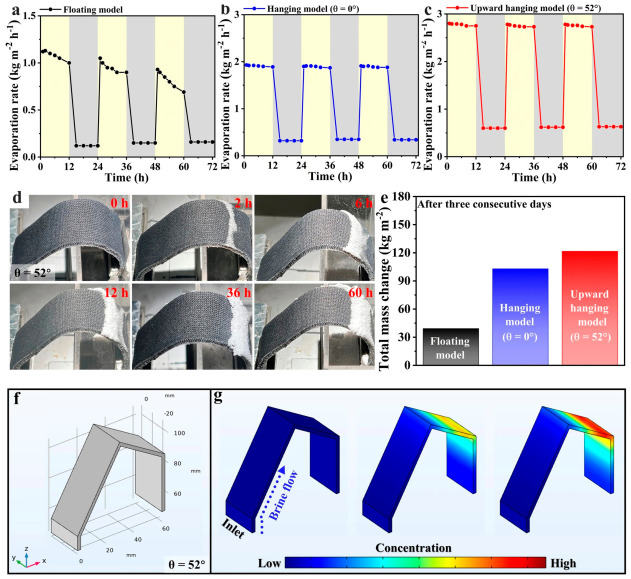
Durability and anti-salt crystallization mechanism of the mangrove-leaf-inspired upward hanging PPy spacer fabric evaporator. (**a**–**c**) Long-term solar evaporation rate profiles of the floating model, horizontal hanging model (θ = 0°), and upward hanging model (θ = 52°) over three consecutive days using 7 wt % brine solution, highlighting the efficacy of spatial isolation in maintaining performance. (**d**) Macroscopic photographs of the upward hanging model (θ = 52°) during the desalination process, illustrating directional salt accumulation at the lower segment while keeping the active evaporation zone clear. (**e**) Comparison of the cumulative evaporated mass among the three configurations after three consecutive days of operation. (**f**,**g**) Geometric schematic and COMSOL-simulated distribution of salt ion concentration over the upward hanging model (θ = 52°) during steady-state evaporation, corroborating the capillary-driven salt migration mechanism. Adapted from ref [158]. The specific COMSOL Multiphysics version was not reported in the cited source.

To fundamentally address the issues of reduced light transmittance and blocked evaporation channels caused by high-salinity crystallization, and to achieve zero-liquid-discharge (ZLD) desalination, Peng et al. [158] designed an upward hanging evaporator based on a 3D polydopamine/polypyrrole spacer fabric (PPSF), inspired by the transpiration and salt-secreting mechanisms of mangrove leaves (Figure 8). The PPSF exhibits a remarkably high solar-absorbing efficiency of 97.8%. As comparatively illustrated in Figure 8a–c, while traditional floating models experience significant evaporation degradation over time, and horizontal hanging models (θ = 0°) avoid crystallization but suffer from heat loss via liquid discharge, the upward hanging model at an optimized tilt angle of 52° minimized the heat loss to 0.366 W and achieved a peak evaporation rate of 2.81 kg·m^−2^·h^−1^ when treating 7 wt% waste brine. More importantly, during a continuous 72 h cyclic test, its solar evaporation rate remained highly stable at ~2.71 kg·m^−2^·h^−1^. Dynamic morphological tracking (Figure 8d) and COMSOL simulations of salt ion concentration (Figure 8f,g) clearly elucidated its intrinsic directional crystallization mechanism: salt is transported upward via capillary action and eventually accumulates and crystallizes exclusively at the downward-facing lower segment of the fabric, achieving a complete spatial isolation between the evaporation and crystallization zones. After three consecutive days of operation, the total mass change of water for this system reached 121.68 kg·m^−2^, which is 3.1 times higher than that of the floating evaporator (Figure 8e). This biomimetic hanging and spatial isolation strategy provides a highly promising engineering solution for PPy-based interfaces to simultaneously realize long-term anti-crystallization failure and solid salt recovery when treating hypersaline brine.

Beyond evaporative desalination, the role of PPy in anti-fouling separation membranes and oil/water separation interfaces is highly prominent. Zhan et al. [159] constructed a Janus PPy/melamine foam with self-healing wettability. The system delivered a solar-driven evaporation rate of 1.52 kg·m^−2^·h^−1^ with an energy efficiency of 92.02%, and an oil/water separation efficiency of 99.7%. Critically, impaired wettability could be restored post-illumination, indicating that PPy-empowered interfaces possess both environmental separation capabilities and self-healing characteristics. Baig et al. [160] introduced a PPy/g-C_3_N_4_ photocatalytic layer onto polymeric-ceramic hybrid membranes, achieving an underwater oil contact angle of 159.9°. At a pressure of 8 bar, the pure water flux was 650 LMH, and the separation efficiency for a 100 ppm water-in-oil emulsion exceeded 99%. Although the flux declined from 92 LMH to 50 LMH after 60 min of operation, it recovered to ~90% of the initial flux upon simulated solar irradiation, verifying its light-driven self-cleaning capability. The O-g-C_3_N_4_/PPy composite membrane designed by Khan et al. [161] exhibited a surface water contact angle of ~13 ± 0.69° and an underwater oil contact angle of 153.2 ± 2.14°. The membrane demonstrated a rejection rate exceeding 96% for EBT dye, a salt permeation rate of 82–97%, and a separation efficiency of >99% for vegetable oil, diesel, and petroleum ether emulsions. Furthermore, 96% of the water flux was recovered after 30 min of illumination post-fouling. These results demonstrate that in environmental membrane separation, PPy not only imparts surface conductivity but, through synergy with photocatalytic phases and hydrophilic/oleophobic interfaces, realizes anti-fouling, self-cleaning, and highly selective separation.

An analogous protective logic extends to interfaces integrating pollutant degradation and separation. Yu et al. [162] introduced PPy/CoZn-LDH onto polyester fabrics, constructing a composite interface featuring both photocatalytic and oil/water separation functionalities. Under neutral conditions, the photodegradation efficiency for tetracycline hydrochloride reached 84.1%, while the oil/water separation efficiency was ~98%. This work demonstrates that when PPy is hybridized with environmentally active phases such as layered double hydroxides (LDHs), the surface is no longer restricted to passively blocking pollutants. Instead, it actively participates in the removal of organic pollutants and the separation of complex liquids, thereby advancing the “environmental barrier” into a comprehensive protective mode of “barrier + purification + functional retention”.

Overall, the environmental protection functionalities of PPy-based coatings discussed in Section 4.3 are fundamentally a coupled process integrating interfacial wettability regulation, continuous photothermal conversion, pollution inhibition, and structural stability. For flexible textiles and surface coatings, the synergy between PPy and PDMS, Ag nanoparticles, or g-C_3_N_4_ concurrently enhances hydrophobicity, self-cleaning, UV protection, and environmental durability. In desalination and evaporation systems, the integration of PPy with porous skeletons such as hydrogels, sponges, waste cotton, and filter cotton significantly bolsters anti-salt crystallization capabilities and long-term operational stability. In the domain of oil/water separation and anti-fouling membranes, compositing PPy with environmentally active phases like g-C_3_N_4_ and CoZn-LDH further endows the interfaces with anti-fouling properties, flux recovery, and pollutant photodegradation capabilities. Therefore, compared to traditional barrier coatings that merely emphasize “water/oxygen isolation”, the recent evolutionary trajectory of PPy-based systems is clearly directed toward multi-dimensional, integrated environmental protection interfaces characterized by “anti-wetting/anti-salting/anti-fouling/self-cleaning/sustainable water treatment”.

## 5. Representative Emerging Applications of PPy-Based Coatings

PPy-based coatings have also been extended to a series of emerging functional interfaces, in which their performance is closely associated with the intrinsic electronic structure and coating-level organization of PPy. In antistatic and electromagnetic interference shielding coatings, PPy mainly contributes lightweight and continuous conductive pathways. In flexible sensing systems, variations in the doping state, charge transport, and conductive network of PPy allow external stimuli such as gas, humidity, strain, and pressure to be converted into measurable electrical signals. In electroactive energy-related coatings, the reversible redox activity and ion-coupled charge transport of PPy support charge storage and electrochromic behavior. For biomedical and bioelectrode coatings, PPy provides a soft and conductive interface for electrical communication with biological tissues. These representative applications therefore reflect the functional extension of PPy’s conductivity, redox activity, doping/dedoping behavior, and structural tunability at the coating interface.

### 5.1. Antistatic and Electromagnetic Interference (EMI) Shielding

Distinct from the surface protection rationale detailed in Section 4, which centers on corrosion inhibition, environmental barriers, and interfacial stability, the application of polypyrrole (PPy)-based coatings in antistatic and electromagnetic interference (EMI) shielding depends directly on the charge dissipation and electromagnetic wave attenuation functionalities afforded by their continuous conductive networks. For antistatic surfaces, the critical objective is not the pursuit of exceedingly high electrical conductivity, but rather the maintenance of the material within a resistance regime capable of dissipating surface static charges rapidly and controllably. For EMI shielding interfaces, it is required not only to form stable electron transport pathways but also to effectively attenuate incident electromagnetic waves through processes encompassing reflection, absorption, and multiple internal reflections. Currently, the research focus on PPy-based coatings in this domain has shifted from initially “acquiring conductivity” to “sustaining stable conductivity and shielding during long-term service in lightweight, flexible, wearable, and complex environments”, thereby broadening their application prospects in flexible electronics, functional textiles, and smart protective interfaces.

In the context of antistatic or low-resistance dissipative interfaces, research emphasizes the controllability of film-forming processes, substrate adaptability, and conductive stability. Naysmith et al. [163] integrated green-synthesized Ag nanoparticles (AgNPs) with in situ polymerized PPy to construct AgNP–PPy/linen conductive fabrics. The resulting samples exhibited a low resistance of 37 Ω, demonstrating that the synergy between PPy and metal nanoparticles can significantly reduce the resistance of textile substrates while adhering to green fabrication principles. Liu et al. [164] compared the conductive performance of PPy coatings across six different substrates. The results indicated that under identical processing conditions, polyester-based substrates exhibited the highest conductivity, followed by nylon and wool. Additionally, PPy was uniformly distributed on the different fabric surfaces and demonstrated a certain degree of washing fastness. Beyond textile substrates, the aqueous carboxylated styrene-butadiene rubber (XSBR)/PPy composite latex developed by Yin et al. [89] demonstrated broad film-forming versatility. The coating could be applied via casting, dip-coating, or spray-coating onto planar or curved substrates such as plastics, glass, rubber, and balloons, yielding a conductivity of ~2 × 10^−3^ S·cm^−1^, and was explicitly identified as an effective antistatic material. Focusing on wearable braided cords, Zhang et al. [87] noted that PPy-modified flexible conductive textile components undergo varying degrees of conductivity decay under mechanical forces such as friction, stretching, and bending. This implies that the evaluation of antistatic/conductive coatings must transcend initial resistance values to prioritize conductivity retention under realistic deformation and frictional conditions. Furthermore, Meng et al. [165] introduced dendritic PPy into rigid polyurethane foams to construct conductive pathways, indicating that the antistatic application of PPy is expanding from flexible textile surfaces to broader foam and structural material interfaces.

In the realm of EMI shielding, PPy research highlights lightweight design, multifunctionality, and stability under harsh environments. Zhou et al. [166] grew PPy in situ on aramid nanofibers (ANFs) to fabricate highly stable ANF@PPy films, proving that PPy can establish stable conductive layers on high-strength, heat-resistant fiber skeletons suitable for flexible EMI shielding interfaces operating in complex conditions. The PAN@PPy/MXene film prepared by Wu et al. [167], despite a thickness of merely 55 μm, achieved an EMI shielding effectiveness of 32 dB and a remarkably high normalized specific shielding effectiveness of 17,534.5 dB·cm^2^·g^−1^. It also demonstrated the potential for integrating shielding with Joule heating by rapidly reaching 170.5 °C under a 4 V driving voltage. Guo et al. [168] further enhanced broadband performance in ANF/PPy composite films; the ANF/PPy-4 film, with a thickness of 46 μm, achieved an EMI shielding effectiveness of 35 dB in the 6–26.5 GHz range while exhibiting substantial mechanical strength. The PPy nanotubes (PNTs)/PDMS/cotton fabric composite system engineered by Meng et al. [147] closely aligns with wearable applications: it possessed a conductivity of ~2715.8 S·m^−1^ and a total EMI shielding effectiveness of 28.2 dB in the X-band. It remained stable after ultrasonication, cyclic bending, and washing, and retained excellent conductive and shielding properties after 365 days of storage. Chai et al. [84] utilized a cellulose nanofiber/PPy/MXene composite strategy; the resulting CNF–PPy film exhibited a conductivity of ~3.91 S·cm^−1^ and an average X-band EMI shielding effectiveness of ~28.1 dB. Upon the incorporation of MXene, the average shielding effectiveness increased to ~45.8 dB, maintaining a capability of ~44.7 dB in the 26.5–40.0 GHz band. The hierarchically porous MF@PPy foam constructed by Liu et al. [52] advanced structural design into three-dimensional hierarchies: the material achieved a water contact angle of 142.00°, a conductivity of ~128.2 S·m^−1^, an EMI shielding effectiveness of 55.77 dB, and a specific shielding effectiveness of 19,928.57 dB·cm^2^·g^−1^. Cheng et al. [169] self-assembled ANFs, PPy, and MXene into a “brick–mortar” composite film, achieving a high conductivity of 775.4 S·cm^−1^, an EMI shielding effectiveness of 40.4 dB, and a specific shielding effectiveness of 36,288.6 dB·cm^2^·g^−1^ at a thickness of only 16 μm. This indicates that PPy-based thin-film materials are advancing toward architectures characterized by minimal thickness, high strength, and high absorption coefficients.

**Figure 9:**
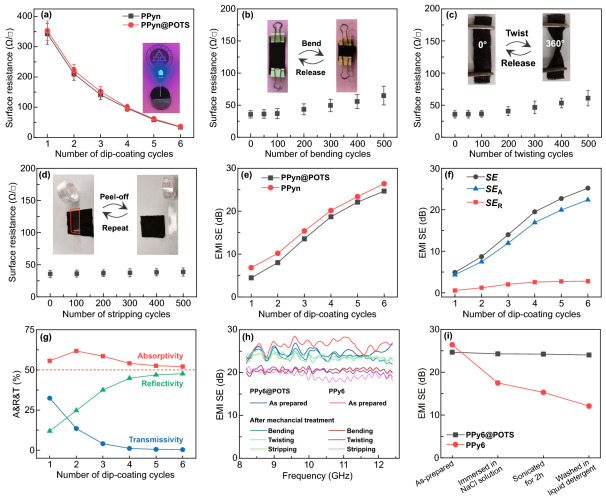
Characterization of the electrical and EMI shielding service stability of PPy-coated fabrics protected by a superhydrophobic POTS layer (PPyn@POTS). (**a**) Surface resistance as a function of PPy dip-coating cycles. (**b**–**d**) Evolution of surface resistance of the PPy_6_@POTS fabric after 500 cycles of mechanical destruction, including bending, twisting, and tape stripping. (**e**–**g**) Trend of EMI shielding effectiveness with increasing coating cycles, along with the corresponding analysis of absorption/reflection effectiveness (SE_A,_ SE_R_) and electromagnetic energy coefficients (absorptivity A, reflectivity R, transmissivity T), highlighting the absorption-dominant characteristics. (**h**,**i**) Pronounced comparison of EMI SE retention between the protected PPy_6_@POTS and unprotected PPy_6_ fabrics following mechanical destruction cycles (500 cycles) and environmental degradation (96 h saline immersion, 45 min washing). Adapted from ref [171].

From an analytical design perspective, the research trajectory for PPy-based antistatic and EMI shielding coatings has shifted from elevating initial conductivity to reinforcing interfacial stability during service and manufacturing scalability. Mahelová et al. [82] established a method for the in situ PPy coating of polyurethane anisotropic electrospun mats, determining that the choice of oxidant and polymerization time concurrently influence the specific conductivity, surface free energy, thickness, and surface morphology of the coating. This underscores the necessity for precise control over the film-forming process. Heredia-Rivera et al. [135] utilized cold atmospheric pressure plasma to directly deposit PPy–Ag nanocomposite layers on conductive fabrics, identifying an optimal precursor mass ratio of 96% pyrrole to 4% AgNO_3_. The resulting coatings, employed as dry electrodes for wearable electrocardiogram (ECG) monitoring, achieved continuous service for approximately 10 days, significantly outperforming the short-term operational mode of traditional gel electrodes. The hierarchical polyimide/PPy/carbon nanofiber composite film constructed by Li et al. [170] further demonstrates that PPy applications are no longer confined to traditional textiles or foams; tunable EMI shielding performance up to 74.59 dB can also be realized on heat-resistant polymer films. To visually demonstrate this operational persistence under severe mechanical and environmental conditions, Zou et al. [171] comprehensively evaluated the electrical and EMI shielding properties of PPy-coated fabrics protected by a superhydrophobic 1H,1H,2H,2H-perfluorooctyltriethoxysilane (POTS) layer (Figure 9). As depicted in Figure 9a, increasing the PPy dip-coating cycles to 6 (PPy_6_@POTS) significantly reduced the surface resistance to 34.3 ± 6.2 Ω/sq, with the ultrathin POTS outer layer imposing negligible negative impacts on the overall conductivity. More importantly, after enduring 500 continuous cycles of bending, twisting, and tape stripping, the composite fabric exhibited only a marginal increase in surface resistance (rising to approximately 65, 61, and 39 Ω/sq, respectively), demonstrating exceptional mechanical adhesion durability (Figure 9b–d). Regarding the electromagnetic attenuation mechanism, the fabric displayed characteristic high-absorption properties (Figure 9e–g): the absorption shielding effectiveness (SE_A_) was vastly superior to reflection, and the electromagnetic absorptivity (A) was consistently maintained above 50%. Furthermore, in stark contrast to the severe performance degradation of unprotected PPy coatings after washing and saline immersion, the PPy_6_@POTS fabric retained a robust EMI shielding effectiveness of ~24 dB even after 500 mechanical damage cycles, 96 h of NaCl solution immersion, or 45 min of machine washing (Figure 9h,i). This study conclusively confirms that for PPy-based EMI shielding systems, the critical determinant of application viability is not merely the static peak shielding value, but the capacity to sustain an intact conductive network and effective electromagnetic attenuation pathways under dynamic deformation, hygrothermal exposure, salt spray, and chemical media via low-surface-energy encapsulation and interfacial reinforcement designs. Overall, the research focus in this field has evolved from basic conductive polymer surface modification to the design of stable interfaces integrating processability, durability, lightweight characteristics, and multifunctional coupling.

In summary, the primary research directions for PPy-based antistatic and EMI shielding coatings can be delineated into three facets: first, enhancing continuous film formation and conductivity retention across various substrates via methods such as in situ polymerization, emulsion coating, and plasma deposition; second, constructing lightweight, highly efficient shielding networks dominated by absorption loss through compositing with constituents like MXene, aramid nanofibers, PDMS, and AgNPs; and third, shifting the evaluation paradigm from initial conductivity or singular static shielding values to the assessment of long-term service stability under conditions of washing, bending, storage, hygrothermal exposure, and complex deformations. Consequently, PPy-based interfaces are now more accurately defined as sustainable conductive/shielding coating systems designed for flexible electronics and complex service environments, rather than merely conventional conductive textile surfaces.

### 5.2. Sensing and Smart Responsive Coatings

Distinct from the functional logic in Section 5.1, which relies primarily on conductive networks for controlled charge dissipation or electromagnetic attenuation, PPy-based sensing and smart responsive coatings are founded upon the coupled conversion between external stimuli and interfacial electrical, morphological, or optical signal variations. This mechanism is inherently predicated on the reversible doping/dedoping behavior of PPy and the charge transport properties of its conjugated backbone. Upon exposure to gas molecule adsorption, mechanical tension/compression, hygrothermal fluctuations, illumination, or chemical environments, the carrier concentration, local conformation, interfacial contact resistance, and volumetric state within PPy undergo modulations, thereby generating measurable resistance, current, strain, or deformation responses (as outlined in Section 2.1). Building on this foundation, recent research on PPy-based functional coatings has focused on achieving higher sensitivity, lower limits of detection (LODs), faster response/recovery times, enhanced cyclic stability, and multi-stimuli coupled responsiveness on flexible substrates and in complex environments.

In gas sensing, because PPy is a p-type conducting polymer with specific sensitivity to reducing gases such as NH_3_, room-temperature ammonia sensing represents a prominent research direction. She et al. [172] fabricated a flexible PPy/silk-fiber ammonia sensor by in situ constructing a PPy layer over a silica nanosphere template introduced onto silk fibers. Operating at room temperature and 68 ± 5% relative humidity, the response to 100 ppm NH_3_ reached 73.25%—significantly higher than the 14.51% of the non-templated control—with a recovery time shortened from 98 s to 69 s. This demonstrates that hierarchical rough structures and high specific surface areas effectively amplify gas adsorption and resistance response signals. Tian et al. [173] coupled MoS_2_ nanosheets enriched with edge sulfur vacancies with an Au-functionalized PPy overlayer to construct a flexible room-temperature ammonia sensing interface. The study indicated that sulfur vacancy active sites, p–n heterojunction effects, and the catalytic activity of Au synergistically enhanced the adsorption and charge transport efficiency of NH_3_ molecules. Cai et al. [174] developed a room-temperature chemiresistive ammonia sensor using a self-assembled PPy/zinc tetraphenylporphyrin (ZnTPP) system, reporting a response to NH_3_ of 104.3%, response/recovery times of 42 s and 223 s, respectively, and an LOD of ~8.63 ppm. The system also achieved wireless signal readout via smartphone integration. Wei et al. [175] proposed a universal strategy for the large-area, controllable fabrication of conductive mesoporous polymer monolayers. The resulting monolayer mesoporous PPy film generated stable responses to 200 ppb NH_3_, with a response time of only 4 s and a recovery time of ~13 s, confirming that integrating a continuous conductive network with an open mesoporous structure satisfies both low LOD and rapid kinetic requirements.

The application of PPy-based gas sensing coatings is extending into biomedical scenarios, such as breath diagnostics. Kamalabadi et al. [176] constructed a PPy/Ag nanoparticle film sensor for detecting NH_3_ in the exhaled breath of COVID-19 patients with acute kidney injury. The device exhibited a linear response from 1.00 to 19.23 ppm, an LOD of 0.12 ppm, and demonstrated a correlation with estimated glomerular filtration rate indices across 19 clinical breath samples. Ferdosi et al. [177] utilized sulfonated reduced graphene oxide-doped PPy films for exhaled ammonia analysis in patients with renal failure. Under room temperature (~26 °C) and high humidity, the sensor yielded two linear response ranges (5–40 ppb and 40–5000 ppb), an LOD as low as 2.7 ppb, and high consistency with clinical blood biochemical outcomes. These studies suggest that evaluation metrics for PPy-based gas sensing coatings are shifting toward low-concentration detection, complex humidity tolerance, wearable integration, and clinical diagnostic relevance.

In strain and pressure sensing, the advantages of PPy stem from systematic variations in conductive pathway continuity, microcrack evolution, hierarchical wrinkled structures, and interfacial contact states during stretching, compression, or bending, enabling the output of stable and amplifiable resistance signals. Researchers have enhanced the stretchability and cyclic durability of PPy by compositing it with elastomers, fibrous skeletons, or 2D conductive components, while concurrently improving sensitivity and low-strain resolution through microstructural design. Muhammad and Kim [65] constructed micro-patterned PPy/PDMS interfaces to fabricate highly sensitive flexible strain sensors, achieving an apparent gauge factor (GF) of ~35 over a 0–100% strain range, a response time of ~2.8 ms for minute strains (0–1%), and sustained stability after 500 cycles. Yan et al. [178] reported a self-healing conductive composite based on PPy and oxidized natural rubber, noting a GF of 3.2 in the low-strain region (<60%) which increased to 477.6 in the high-strain region (>430%). This indicates that coupling a self-healing elastic network with a PPy conductive phase balances large deformation adaptability with high-sensitivity responses. Gao et al. [179] utilized Ecoflex to encapsulate a bacterial cellulose/PPy structure, yielding a biodegradable flexible strain sensor with an LOD of 0.05%, a working strain range of 90%, and a GF between 3.21 and 4.86, capable of stable motion monitoring after 1000 cycles at 90% strain.

To construct higher-performance wearable sensing interfaces, the synergistic effects of PPy with MXene, metal nanoparticles, and textile skeletons have been extensively exploited. Wang et al. [180] prepared hierarchical PPy@MXene fiber sensors exhibiting a sensing range of 0–106% and a stretchability exceeding 750%, with the GF surging from 60 to 3.23 × 10^6^ across different strain regimes. This verifies that coupling hierarchical fibrous conductive networks with 2D nanosheets significantly amplifies deformation-induced resistance modulations. Peng et al. [181] engineered a superhydrophobic fabric sensor based on an AgNPs/PPy conductive network, achieving a GF of 1.61 × 10^3^ in the 60–70% strain range, a response time of ~70 ms, and cyclic stability over 5000 cycles. The surface water contact angle of ~156° demonstrated excellent resistance to moisture perturbation. Li and Huang [182] coated PPy onto a TPU hierarchical array to construct a multifunctional flexible sensing interface capable of both pressure and gas response. The device yielded a pressure sensitivity of 7.2 kPa^−1^ in the low-pressure regime (0–800 Pa), an LOD of 6.2 Pa, and maintained stability over 10,000 cycles. Coupled with early studies by Yang et al. [183] on three-scale nested wrinkling PPy film pressure sensors, it is evident that enhancing PPy strain/pressure sensing performance fundamentally relies on establishing highly deformation-sensitive, structured resistance modulation pathways via controlled wrinkling, hierarchical cracking, flexible skeletons, and multiphase interfaces.

**Figure 10:**
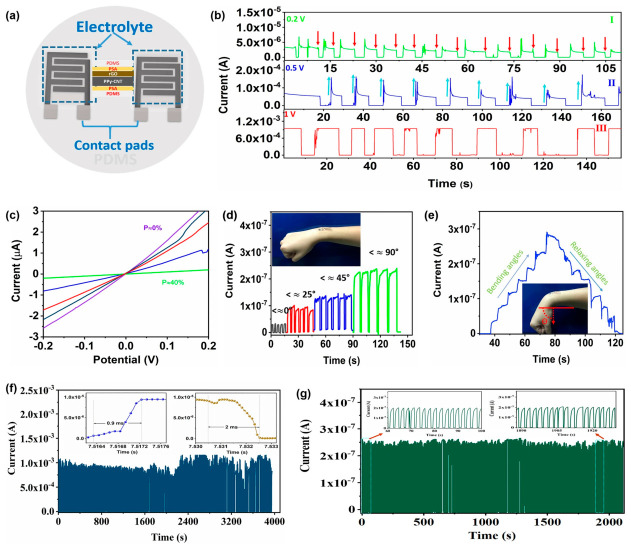
Electromechanochemical performance characterization of the flexible micro-strain sensor derived from the PPy-CNT@rGO microsupercapacitor (MSC). (**a**) Schematic diagram of the interdigital flexible micro-strain sensor. (**b**,**c**) Dynamic current responses and current-voltage curves of the device under various applied strains (0–40%) at different driving voltages (0.2, 0.5, 1 V). (**d**,**e**) Step-wise current response and relaxation monitoring of the sensor during continuous wrist bending from 10° to 90°. (**f**) Cyclic durability test of the device over 2500 stress/release cycles at an applied voltage of 1.0 V and a peak stress of 50 kPa; the inset displays an ultrafast response time of 0.9 ms and a recovery time of 2 ms. (**g**) Durability test over 800 bending/release cycles at a maximum bending angle of 90°. Adapted from ref [184]. In panel (**b**), I, II, and III correspond to the current responses measured at 0.2, 0.5, and 1.0 V, respectively; the arrows indicate strain/release events during dynamic testing. In panel (**c**), the differently colored curves represent current–voltage responses under different applied strain levels from 0% to 40%.

To address the susceptibility to short-circuiting and the sluggish response inherent in traditional sandwich-structured capacitive sensors, the integration of planar interdigital architectures with iontronic mechanisms provides a viable pathway for flexible sensing. Tahir et al. [184] developed a current-collector-free flexible micro-strain sensor derived from a PPy-CNT@rGO microsupercapacitor (MSC) (Figure 10). As depicted in Figure 10a, the interdigital microelectrodes were transferred onto a polydimethylsiloxane (PDMS) flexible substrate; under applied stress, the gel dielectric layer fills the inter-electrode channels, and the deformation of the porous PPy-CNT network increases the effective contact interface between the active material and the electrolyte, thereby outputting dynamic current signals by modulating the pseudocapacitance and electric double-layer capacitance. Electrical testing indicated that under driving voltages of 0.2 to 1 V, the sensor generated systematic current step responses to strains up to 40% (Figure 10b,c); in wrist bending tests, the device accurately resolved step-wise angular variations from 10° to 90° (Figure 10d,e). Kinetic and durability characterizations (Figure 10f,g) revealed that the response and recovery times of the sensor were only 0.9 ms and 2 ms, respectively, and it maintained extremely high signal output consistency after enduring 2500 compression cycles at a peak stress of 50 kPa and 800 bending cycles at a maximum of 90°. This design, which directly translates a micro-energy storage unit into a highly sensitive electromechanochemical sensing interface, further substantiates the exceptionally high engineering reliability of PPy-based composite networks in the continuous acquisition of minute physiological signals, such as pulse monitoring, swallowing movements, and respiration.

In the domain of smart responsive surfaces, PPy-based coatings exhibit a trajectory toward integrated “sensing-actuation-adaptive deformation”. Wang et al. [185] constructed high-performance multi-responsive bilayer actuators based on micro/nanostructured PPy, demonstrating that PPy can actuate interfacial deformation under thermal, optical, or chemical stimuli, realizing active responsive behavior. Yim et al. [186] fabricated a controllable porous membrane actuator based on PVDF and PPy through gradient infiltration of conducting polymers. This device translates external stimulation into controllable bending deformation, displaying potential in soft actuation and smart devices, thereby validating PPy’s reliability as an active responsive layer. Liu et al. [187] integrated conductive PPy nanofibers into a poly(N-isopropylacrylamide) (PNIPAM) hydrogel to construct a thermally and optically dual-driven soft actuator. The system featured NIR responsiveness, rapid shape recovery, and strain self-sensing capabilities, signifying that PPy functions as a core responsive phase in multi-field coupled soft devices. Furthermore, the PPy/agar nanocomposite bidirectional bending actuator developed by Wang et al. [188] exhibited synergistic responses to stimuli including humidity, light, temperature, HCl, and NH_3_. This indicates that integrating PPy with stimuli-responsive matrices expands interfacial functionalities from singular sensing to programmable, multi-stimuli responsive smart surfaces.

In summary, the progression of PPy-based sensing and smart responsive coatings reflects a functional evolution from passive signal detection to high-sensitivity, multi-stimuli coupling, and integrated active actuation. For gas sensing, future imperatives involve enhancing specific gas selectivity, mitigating humidity interference, and ensuring long-term stability in complex exhaled breath or environmental atmospheres. For strain/pressure sensing, optimizing the balance among sensitivity, deformation range, response speed, and fatigue durability remains essential. Regarding stimuli-responsive smart surfaces, the developmental potential of PPy lies in its profound integration with elastomers, hydrogels, 2D materials, and microstructured substrates. Sustaining stable doping states, precisely regulating microstructures, preserving continuous conductive pathways, and ensuring interfacial reliability under complex environments remain the foundational prerequisites for constructing high-performance PPy smart responsive interfaces. In addition, advanced signal-processing and physics-informed deformation-measurement methods may further improve the reliability of PPy-based wearable sensing systems by enhancing motion-artifact removal, strain-field reconstruction, and long-term signal interpretation [189,190,191].

### 5.3. Electroactive and Energy-Related Coatings

Distinct from the sensing interfaces in Section 5.1, which rely on conductive networks for static dissipation and electromagnetic attenuation, and those in Section 5.2, which center on stimuli responsiveness, the functionality of PPy-based electroactive and energy-related coatings depends directly on their reversible redox behavior, doping/dedoping processes, and synergistic ion-electron transport capabilities. In these systems, PPy functions not only as a conductive phase but simultaneously acts as a pseudocapacitive active layer, a charge transport channel, and an interfacial electrochemical response unit. Consequently, these coatings are highly applicable for constructing supercapacitor electrodes, electrochromic films, and integrated energy storage-electrochromic functional interfaces. Recently, the research focus in this domain has transitioned from merely pursuing high specific capacitance to comprehensively optimizing energy storage performance, cyclic stability, mechanical compliance, and optical feedback capabilities under flexible, lightweight, and visualizable conditions [173,174,175,176,177,178,179,180,181,182,183,184,185,186,187,188]. In the field of electrochromic energy storage devices, because charge storage and color modulation share similar redox mechanisms, PPy and its composite systems are extensively utilized in the development of electrochromic supercapacitors and energy storage electrochromic devices.

Regarding supercapacitor electrodes, PPy-based coatings can construct continuous conductive networks via surface coating, in situ polymerization, or hierarchical compositing, thereby significantly enhancing the pseudocapacitive contribution on flexible substrates [52,168,169,170,171,172]. Recently, this strategy has been widely validated in paper-based, fiber-based, and freestanding films. Fan et al. [192] constructed a high-loading flexible paper-based PPy electrode, emphasizing the combination of self-assembly processes with high-loading active layers to realize high-performance flexible supercapacitor interfaces. Dang et al. [193] utilized electrodeposition to fabricate a graphene/PPy electrode, achieving a “large areal capacitance” flexible supercapacitor, which verified the applicability of electrodeposition in constructing thin-layer PPy composite electrodes. The Ti_3_C_2_T_x_/TPU/PPy fiber electrode prepared by Zhang et al. [194] combined flexibility with weavability; this fiber electrode exhibited a specific capacitance of 41.2 F·g^−1^ at 5 mV·s^−1^, and the symmetric fiber supercapacitor assembled from it delivered an energy density of 2.36 mWh·g^−1^, with a capacitance retention of ~90.39% after 10,000 charge/discharge cycles. The freestanding rGO/PPy film reported by Zhu et al. [195] demonstrated potential for wearable applications: the symmetric device exhibited an areal capacitance of 631 mF·cm^−2^, a volumetric capacitance of 117,000 mF·cm^−3^, and an areal energy density of 56.1 μWh·cm^−2^, retaining 86% of its initial capacitance after 800 bending cycles. Sun et al. [196] utilized SnCl_2_-modified bacterial cellulose as a skeleton to deposit PPy via electrostatic self-assembly, obtaining a flexible electrode with an areal capacitance up to 5718 mF·cm^−2^; the electrode maintained a capacitance retention of 86.8% after 10,000 cycles, with no significant performance degradation under various bending states. Arena et al. [197] applied an aqueous PPy ink directly to paper-based solid-state supercapacitors, demonstrating that even a low-cost and straightforward “drawn-on-paper” route can achieve areal capacitances on the order of 100 mF·cm^−2^, providing a viable pathway for printable electroactive coatings.

Beyond enhancing capacitance, another developmental trend for PPy-based energy-related interfaces is deep coupling with multi-field responsiveness and flexible structures, evolving the electrode from a singular “energy storage layer” to an electrochemical functional surface possessing mechanical adaptability, environmental responsiveness, and self-sensing capabilities. The PPy/hydrogel hybrid film system proposed by Shabeeba et al. [198] was defined as “multi-sensing supercapacitor electrodes”, indicating that PPy-based electrodes can synchronously perceive electrical, thermal, and chemical perturbations during charge/discharge processes, reflecting an expansion toward “smart electrode interfaces”. Analyzing the logic of interfacial design, such performance improvements do not stem solely from an increase in PPy content but rely on the synergistic effects among porous networks, fibrous skeletons, 2D nanosheets, and gel/paper-based supporting materials. High-specific-surface-area structures help shorten ion diffusion pathways; flexible skeletons buffer the volume expansion and mechanical embrittlement of PPy during long-term cycling; and conductive components like graphene and MXene further optimize electron transport efficiency and overall structural stability [174,175,176,177]. Thus, the function of PPy-based coatings in supercapacitors has evolved from being solely an active conducting polymer layer in early studies to acting as an interfacial regulatory center within multiscale composite electrodes [173,174,175,176,177,178,179].

**Figure 11:**
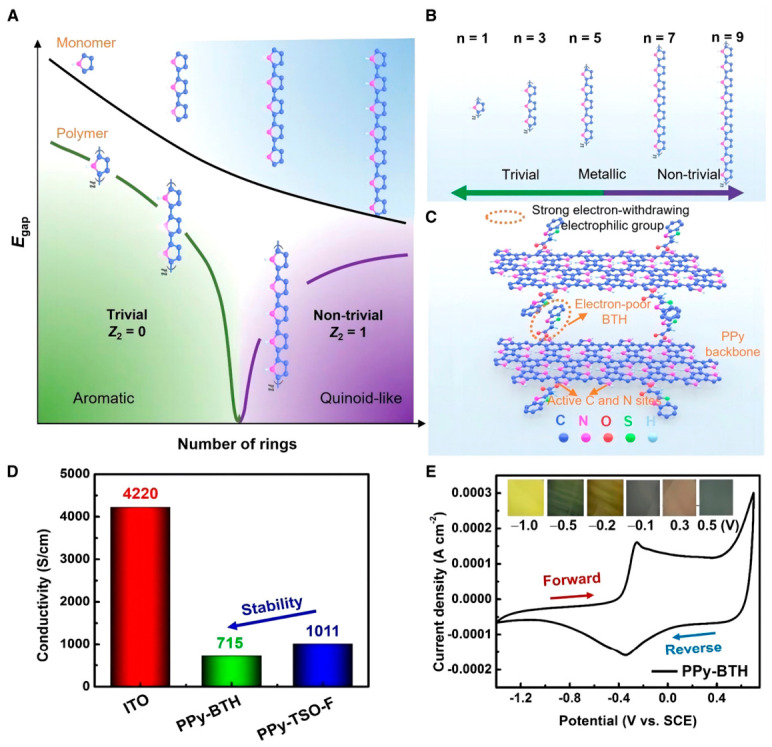
Molecular design and electrochromic performance of the narrow-bandgap “quasi-metallic” PPy derivative (PPy-BTH) with tunable electronic structures. (**A**,**B**) Evolution model of the energy gap (Egap) as a function of the number of rings, illustrating the topological phase transition from a trivial aromatic state (Z_2_ = 0) to a gapless metallic state and subsequently to a non-trivial quinoid-like state (Z_2_ = 1). (**C**) Molecular structure of PPy-BTH highlighting the modulation of the PPy backbone via electron-poor BTH units and strong electron-withdrawing groups. (**D**) Electrical conductivity of PPy-BTH compared with reference materials. (**E**) Cyclic voltammetry (CV) curve of the PPy-BTH film alongside its corresponding multi-color electrochromic states at low driving voltages (−1.0 V to 0.5 V). Adapted from ref [201].

In the realm of electrochromism, the application of PPy is similarly founded on the electronic structural changes and optical absorption modulations induced by reversible redox reactions. Ratautaite et al. [199] systematically investigated the electrochromic behavior of a PPy/poly(methylene blue) composite layer. The study indicated that under pulse potentials of +0.8 V/−0.8 V, the composite layer exhibited significant optical absorption changes at both 668 nm and 750 nm, with the most intense response occurring in acidic media. Research by Lou et al. [200] demonstrated that the polymerization process critically determines the final performance of PPy electrochromic films. They prepared PPy films on FTO conductive glass using alternating current (AC) electrochemical impedance spectroscopy; when the AC amplitude was controlled at 100 mV, the resulting film displayed a uniform nanosphere morphology, achieving a coloration efficiency of 137.4 cm^2^·C^−1^, coloration/bleaching times of 5.0 s and 6.5 s, and retaining 65.7% of its light modulation capability after 100 cycles. Recently, Gao et al. [201] constructed a narrow-bandgap “quasi-metallic” PPy derivative (PPy-BTH) by introducing benzo-2,1,3-thiadiazole (BTH) acceptor units into the PPy backbone (Figure 11). As illustrated in the energy gap evolution model (Figure 11A,B), increasing the number of repeating units drives the polymer from a topologically trivial aromatic phase (Z_2_ = 0) toward a gapless metallic state, eventually transitioning into a non-trivial quinoid-like phase (Z_2_ = 1). This rational molecular design (Figure 11C) leverages the electron-poor BTH and strong electron-withdrawing groups to precisely modulate the electronic structure of the PPy backbone. This material possessed a bandgap of merely 0.35 eV, a carrier mobility of 32.5 cm^2^·V^−1^·s^−1^, and a remarkable macroscopic electrical conductivity of 715 S·cm^−1^ (Figure 11D). Benefiting from these quasi-metallic characteristics, the cyclic voltammetry (CV) redox peaks of the PPy-BTH film are tightly coupled with its multi-color electrochromic transitions—e.g., from yellow at −1.0 V to dark green/gray at 0.5 V (Figure 11E)—realizing high-contrast dynamic color changes and high-saturation visual outputs driven by low voltages. This indicates that the electrochromic performance of PPy has progressed beyond traditional unmodified pyrrole systems, advancing toward high-performance electrochromic polymers with designable molecular structures and precisely tunable bandgaps.

Furthermore, PPy-based electroactive interfaces are becoming essential components of coupled energy storage-electrochromic devices. The PPy/Prussian Blue (PB) bilayer electrochromic device proposed by Ma et al. [202] confirmed that constructing a potential difference-driven alternating redox process at the solid/solid/liquid interface significantly enhances the self-coloration and self-recharging capabilities of the device. The core mechanism involves the reduced state of PPy being oxidized by PB, while the generated Prussian white can be re-oxidized by dissolved oxygen in the solution, thereby establishing a continuous, self-recovering electron transfer pathway. From the macroscopic trend of device development, electrochromic supercapacitors (ECSCs) and energy storage electrochromic devices (EESDs) have become research hotspots in the field of conducting polymers [186,187,188]. Relevant literature identifies the main challenges for such devices as balancing high specific capacitance with high optical contrast, improving device flexibility and stretchability, and achieving precise, quantitative visual correlation between color changes and the state of charge (SOC). Due to the natural coupling of its intrinsic pseudocapacitance and reversible colorimetric response, PPy remains a core candidate material in this direction, though it must overcome issues related to volumetric strain during cycling, long-term stability degradation, and limited color richness.

In addition to supercapacitors and electrochromic devices, PPy-based energy-related coatings are extending into broader electrochemical energy interfaces [184,185]. Han et al. [203] in situ polymerized PPy on the surface of a Prussian blue analog cathode to construct an MZHCF@PPy composite for high-capacity flexible zinc-ion batteries. The material delivered a specific capacity of 190.1 mAh·g^−1^ at a current density of 0.5 A·g^−1^, approximately three times that of the uncoated material, and maintained stable energy storage performance under deformation states such as bending. This suggests that the PPy coating plays a positive role in inhibiting structural degradation of the cathode and enhancing ion adsorption. Dadashi et al. [204] prepared a PPy-Cu_2_O-MoO_3_ ternary nanocomposite layer on a graphene oxide/graphite felt electrode (GO/GFE) via a one-step electrochemical deposition method, enabling the same electrode to be applied simultaneously for supercapacitors and the hydrogen evolution reaction (HER). The electrode exhibited an areal capacitance of 1010.30 mF·cm^−2^ in 0.5 M H_2_SO_4_; the symmetric solid-state device built from it delivered a capacitance of 596.5 mF·cm^−2^ at 1 mA·cm^−2^, with an 82.4% capacity retention after 6000 cycles. In HER catalysis, it exhibited an overpotential of 361 mV at a current density of 10 mA·cm^−2^, with a Tafel slope of 142 mV·dec^−1^. This confirms that the application of PPy coatings in the “energy-related” domain has expanded from traditional energy storage interfaces to dual-functional energy storage-catalysis electrodes.

Overall, the evolution of PPy-based electroactive and energy-related coatings delineates three primary trajectories: first, flexible pseudocapacitive electrodes utilizing paper, fiber, and film substrates, which optimize areal capacitance, rate performance, and bending stability via porous structures and composite conductive networks; second, electrochromic films and energy storage-colorimetric interfaces based on reversible redox reactions, enabling the optical visualization of charge storage states; and third, expansion into generalized electrochemical energy interfaces, such as cathode protection in zinc-ion batteries and electrocatalytic hydrogen evolution, marking the evolution of PPy from a simple “conducting polymer coating” to an “electroactive thin layer combining interfacial regulation, energy storage, and energy conversion functions”. Future research priorities in this direction include mitigating the volume expansion and structural degradation of PPy during long-term cycling; improving ion transport efficiency within thick-film electrodes; achieving a broader range of optical modulation and higher color designability at low driving voltages; and accelerating practical engineering applications in wearable energy storage devices, smart optical windows, and multifunctional electrochemical interfaces. Recent work on solid-state electrolyte interfaces also provides useful inspiration for stabilizing ion transport and interfacial compatibility in future PPy-based flexible electroactive coatings [205].

### 5.4. Biomedical and Biointerface Applications

Distinct from the conductivity-derived applications centered on static dissipation and electromagnetic attenuation in Section 5.1, and the stimuli-responsive and electrochemical functional interface designs in Section 5.2 and Section 5.3, the application of polypyrrole (PPy)-based coatings in biomedical and biointerface fields emphasizes their direct interactions with bacteria, cells, tissues, and electrophysiological signals. In this context, the utility of PPy lies not only in its intrinsic conductivity but also in its positively charged doped backbone, tunable surface energy, robust electrochemical activity, and excellent interfacial compatibility with biological or functional components such as collagen, bacterial cellulose, hydrogels, metal nanoparticles, and bioactive molecules. These attributes enable PPy to serve in diverse scenarios, including antibacterial surfaces, wound healing interfaces, implant coatings, and soft bioelectrodes [188,189,190,191,192,193,194,195,196,197,198,199,200,201,202,203]. Recent research trends indicate that PPy-based biointerfaces have transitioned from exhibiting “singular antibacterial or conductive” properties to adopting multifunctional synergistic designs encompassing “antibacterial activity, immunomodulation, tissue repair, and low-impedance signal acquisition”. Antibacterial surfaces and bioelectrodes represent the two most prominent trajectories within this domain.

**Figure 12:**
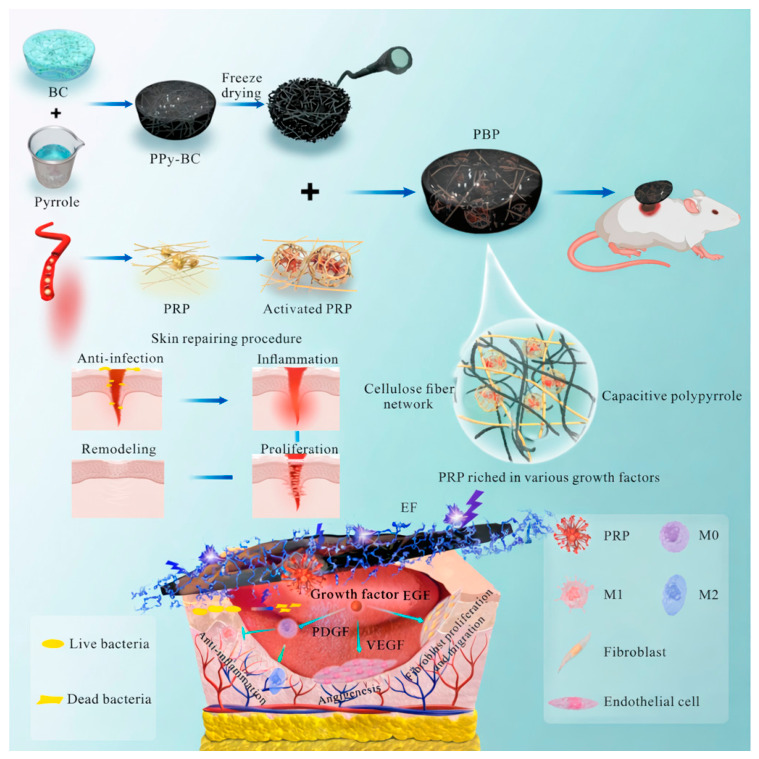
Fabrication process and active therapeutic mechanism of the PBP (polypyrrole/bacterial cellulose/platelet-rich plasma) composite hydrogel designed for diabetic wound repair. Top: Schematic of the assembly process, involving the construction of a porous PPy-BC capacitive skeleton via in situ polymerization and freeze-drying, followed by PRP loading. Bottom: The micro-pathways of the dressing throughout the four stages of skin repair (anti-infection, inflammation regulation, cell proliferation, and remodeling). Under the synergy of an applied electrical field (EF), the active interface not only directly eradicates free bacteria but also facilitates the release of bioactive molecules, including epidermal growth factor (EGF), platelet-derived growth factor (PDGF), and vascular endothelial growth factor (VEGF). This process subsequently induces macrophage polarization (M0 to M1/M2) and accelerates the proliferation of fibroblasts and endothelial cells, alongside angiogenesis. Adapted from ref [207].

In the realm of antibacterial surfaces, PPy research has progressed from qualitative descriptions of its inherent bacteriostatic properties to the development of functionalized antibacterial interfaces integrating electrical stimulation, photothermal conversion, nitric oxide (NO) release, microneedle delivery, and wound microenvironment regulation. Piccioni et al. [67] demonstrated that the antibacterial efficacy of PPy-coated fabrics is highly dependent on monomer concentration, substrate properties, and contact time. For cotton and polyamide 6.6 (PA6.6) fabrics coated with 2 g/L pyrrole, notable bactericidal effects against *Staphylococcus aureus* (*S. aureus*) and *Escherichia coli* (*E. coli*) were observed within 30 min; however, both conductivity and antibacterial activity were susceptible to washing-induced dedoping. Wang et al. [206] engineered a capacitive antibacterial dressing composed of PPy-wrapped carbon cloth electrodes and a bacterial cellulose hydrogel separator. Under 1 V electrical stimulation for 10 min, the bactericidal efficiency reached 99.97% against typical bacteria and 99.99% against multidrug-resistant strains. Driven by its substantial capacitance and rechargeability, the system achieved continuous sterilization. Subsequently, the same group [207] integrated PPy, bacterial cellulose, and platelet-rich plasma (PRP) to develop a PBP composite hydrogel tailored for chronic wound repair. To comprehensively elucidate the active therapeutic mechanism of this dressing in chronic wounds, Wang et al. [207] detailed the fabrication process and in vivo functional pathways of the PBP hydrogel (Figure 12). As depicted in the upper section of Figure 12, this dressing is constructed by polymerizing capacitive PPy within a bacterial cellulose (BC) network to form a porous skeleton, which is subsequently freeze-dried and loaded with activated PRP. Throughout the overarching procedure of skin repair, the material systematically intervenes in four stages: anti-infection, inflammation regulation, cell proliferation, and tissue remodeling. The mechanistic model (Figure 12, lower section) reveals that under the synergistic effect of an applied electrical field (EF) and the capacitive properties of PPy, the composite interface not only directly electrocutes free bacteria but also facilitates the sustained release of diverse bioactive molecules from the PRP, including epidermal growth factor (EGF), platelet-derived growth factor (PDGF), and vascular endothelial growth factor (VEGF). This system exhibited enhanced bactericidal capability, promoted the proliferation of fibroblasts and endothelial cells, and modulated macrophage polarization. In diabetic wound models, it significantly reduced inflammation and accelerated angiogenesis and collagen deposition. These findings indicate that in wound antibacterial scenarios, PPy has evolved from a passive bactericidal surface into an active interface capable of modulating both infection and healing processes under electrical stimulation or charge-storage conditions.

Furthermore, the photothermal and electroactive properties of PPy facilitate its coupling with other therapeutic mechanisms, forming enhanced biointerfaces for complex infected wounds. Guo et al. [208] constructed a PP-Mo-NO polypyrrole hydrogel combining near-infrared (NIR) photothermal effects with NO release. Under 808 nm, 1.0 W·cm^−2^ irradiation for 10 min, the system at a concentration of 100 μg·mL^−1^ exhibited bactericidal rates exceeding 99.1% against *E. coli* and *S. aureus*, with a photothermal efficiency of 79.88%, effectively promoting the repair of infected wounds. In another study, Guo et al. [209] integrated α-amylase and polydopamine@PPy into hydrogel microneedles. Upon NIR irradiation, the microneedle temperature stably increased to ~50 °C, achieving bactericidal rates of 99% and 98% against planktonic *S. aureus* and *E. coli*, respectively, and disrupting 83.2% of *S. aureus* biofilms. The system also exhibited a 73.35% DPPH radical scavenging capability. Nie et al. [210] utilized PPy in a human basic fibroblast growth factor (hFGF2)-oil body system, leveraging its photothermal effect to suppress postoperative melanoma residue and recurrence, while concurrently accelerating wound healing via hFGF2 release. This expands the function of PPy biointerfaces from “antibacterial and repair” to composite scenarios of “postoperative local tumor therapy + wound recovery”. Collectively, recent PPy antibacterial surfaces exhibit a clear trend toward multi-mechanism synergy—encompassing photothermal, electrical stimulation, NO release, and growth factor regulation—rather than being confined to singular contact killing.

For implant-related biointerfaces, the antibacterial function of PPy is further coupled with requirements for corrosion resistance, cytocompatibility, and osseointegration. Mayouf et al. [211] utilized double-pulse electrodeposition to rapidly deposit Ag nanoparticles (AgNPs) onto ~4 μm thick PPy films to enhance conductivity and antibacterial activity, demonstrating the broad applicability of the “PPy-based conductive layer + metal nanoparticles” model in antibacterial biosurfaces. Luo et al. [115] constructed an AgNPs-composited co-doped PPy/Zn coating on ZK60 magnesium alloy, addressing the integrated anti-corrosion and antibacterial requirements for biodegradable metal implants. Xu et al. [78] proposed a strategy involving the cathodic deposition of a PPy/dicalcium phosphate dihydrate (DCPD) composite layer, noting that PPy, as a pre-conductive layer, not only improved the corrosion resistance of magnesium implants but also provided a stable interface for the subsequent deposition of bioactive calcium phosphate (CaP) phases, thereby simultaneously enhancing cytocompatibility and osseointegration potential. Furthermore, Sahoo et al. [63] fabricated a stearic acid-treated PPy superhydrophobic coating on Mg-Ce alloys. With a water contact angle of ~153°, the coating improved corrosion resistance by over 85%, achieved antibacterial efficiencies > 90% against *E. coli* and *S. aureus*, and demonstrated superior osseointegration and fracture healing in rabbit femur and goat tibia models. Consequently, in implant-oriented applications, the role of PPy has transformed from a mere conductive polymer coating into a critical component of a multifunctional interface integrating corrosion protection, antibacterial activity, cytocompatibility, and tissue integration.

Beyond antibacterial and implant interfaces, the application of PPy in bioelectrodes is primarily predicated on its low interfacial impedance, excellent mechanical compliance, and electrochemical characteristics matched to soft tissues. Zhao et al. [212] fabricated a PPy-leather dry electrode for long-term electroencephalogram (EEG) monitoring. The study showed that when worn on the forehead, the interfacial impedance stabilized at ~15 kΩ, which is below the 20 kΩ threshold typically required for EEG electrodes. Its high breathability and sweat tolerance enabled prolonged continuous wear, reliably recording electrooculogram signals and alpha wave activity. The dopamine-PPy/poly(vinyl alcohol) (DA-PPy/PVA) anisotropic hydrogel constructed by Chen et al. [213] exemplified the advantages of PPy in flexible hydrogel bioelectrodes: the system maintained a linear response up to 400% strain with a gauge factor (GF) of 3.00 in the parallel direction, and the acquired electrocardiogram (ECG) and electromyogram (EMG) signals were superior to those of commercial electrodes. Guan et al. [214] utilized a nanocellulose template to induce a PPy electro-osmotic flow network, simultaneously achieving a tissue-matching modulus of 288 kPa and a high electrical conductivity of 135.75 S·m^−1^ at a low percolation threshold. The resulting hydrogel electrode exhibited lower interfacial impedance, superior charge storage and injection capacities, and a higher signal-to-noise ratio for physiological signals compared to commercial electrodes. These results indicate that PPy-based soft electrodes are not merely “conductive” but effectively balance mechanical softness, adhesiveness, conductivity, and low-impedance contact.

**Figure 13:**
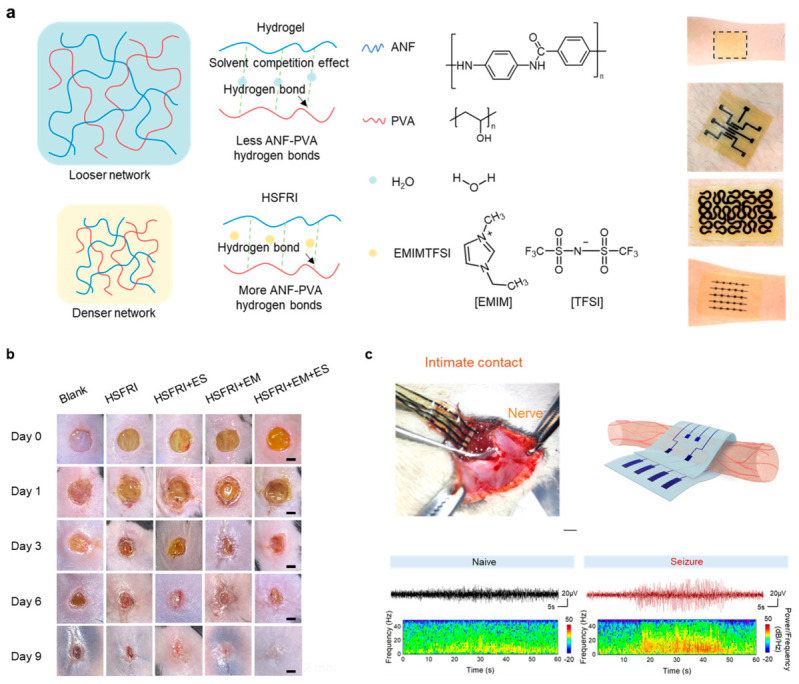
Fabrication and applications of PPy-integrated biogel bioelectronics. (**a**) Schematic illustration of the design and seamless integration of PPy into an ionogel matrix [215]. (**b**) Application of the PPy-integrated ionogel electrode for electrically stimulated wound healing. (**c**) Application of ultrathin PPy-integrated hydrogel electrodes for neural bioelectronics [216]. The dashed boxes indicate selected device/contact regions shown or magnified in the original source figure.

The integration of polypyrrole (PPy) into soft gel matrices, such as hydrogels and ionogels, stands out as a profoundly transformative paradigm for next-generation bioelectronic interfaces. By synergistically combining the intrinsic electroactivity of PPy with the tissue-like compliance, exceptional biocompatibility, and remarkable tunability of soft matter, this strategy effectively dissolves the long-standing dichotomy between rigid electronics and dynamic biological systems, opening an entirely new design space for seamless human–machine integration. In a striking and highly innovative study, Zhang et al. masterfully harnessed solvent-regulated microphase interactions to construct a robust soft–hard interfacial architecture within ionogels, achieving an unprecedented balance between mechanical strength and toughness—an accomplishment that pushes the limits of soft materials engineering (Figure 13a) [215]. Through an elegantly designed in situ polymerization strategy, PPy was integrated in a truly interface-free manner onto the ionogel surface, enabling efficient functionalization while preserving structural continuity. Remarkably, this ionogel platform is fully compatible with ultrathin film fabrication, allowing conformal, three-dimensional integration with biological tissues (Figure 13b). When coupled with electrical stimulation, the system demonstrates rapid and effective wound healing, representing a compelling breakthrough in bioelectronic therapeutics. In a parallel advance, Zhang and co-workers further extended this concept by developing ultrathin PPy–hydrogel electrodes capable of high-fidelity neural stimulation and recording, with successful application in intraoperative epilepsy monitoring (Figure 13c) [216]. These achievements collectively redefine the design principles of bioelectronic interfaces, establishing a new benchmark for softness, functionality, and integration. Collectively, these works constitute a breakthrough advance in the field of bioelectronic interfaces, providing new paradigms for the development of high-performance and multifunctional bioelectronic systems.

In the deeper context of implantable bioelectrode interfaces, recent PPy research has shifted toward immune compatibility and long-term in vivo stability. Lee et al. [217] constructed an interleukin-4 (IL-4)-immobilized PPy/heparin (Hep) electrode, demonstrating that the interface induces macrophage polarization toward an anti-inflammatory phenotype, reduces scar formation, and sustains highly sensitive in vivo ECG recording for up to 15 days. Subsequently, the same research vector led to the development of a hemin-conjugated heparin-doped PPy/HepH electrode [218]. This system possessed catalase-like reactive oxygen species (ROS) scavenging capabilities, reducing intracellular ROS, inhibiting inflammatory macrophage polarization, and extending the duration of high-quality in vivo ECG monitoring to 20 days. Regarding neural electrode interfaces, Wu et al. [219] proposed a collagen/PPy composite film modification strategy. The interface provides a biomimetic biochemical microenvironment via collagen, improves the electrochemical performance of the electrode via PPy, and mitigates neuroinflammation at the electrode interface by inhibiting the inflammatory activation of astrocytes through appropriate electrical stimulation. This series of works illustrates that PPy bioelectrode research has evolved from an early focus on impedance reduction and signal enhancement to the design of “immune-friendly electrode interfaces” capable of actively regulating ROS, inflammation, and glial responses.

Overall, the evolutionary trajectory of PPy-based biomedical and biointerface applications reflects two distinct directions. On the one hand, in antibacterial surfaces, PPy is upgrading from a “conductive layer with certain bacteriostatic properties” to an “active therapeutic interface coupled with electrical stimulation, photothermal conversion, NO release, microneedle delivery, and growth factor regulation”. On the other hand, in bioelectrodes, PPy is transitioning from a “low-impedance conducting polymer” into a “soft interfacial material possessing tissue-modulus matching, long-term signal stability, and immunomodulatory capabilities” [188,189,190,191,192,193,194,195,196,197,198,199,200,201,202,203]. Therefore, compared to traditional medical surfaces that pursue either singular antibacterial or conductive properties, PPy-based biointerfaces are more aptly characterized as multifunctional, dynamic interfacial systems oriented toward the synergistic optimization of infection control and biological integration.

Overall, the emerging applications discussed in this section demonstrate that PPy-based coatings are most valuable when their intrinsic conductivity, redox activity, and structural tunability are directly translated into coating-level functions. In antistatic and EMI shielding coatings, PPy provides continuous and lightweight conductive pathways. In sensing coatings, PPy converts external stimuli into measurable electrical or optical responses through doping/dedoping and conductive-network modulation. In electroactive energy-related coatings, PPy contributes reversible redox activity and ion-coupled charge storage. In biomedical and bioelectrode coatings, PPy provides a soft and conductive interface for electrical communication with biological tissues. Thus, these applications are better understood as representative extensions of PPy’s coating-specific advantages rather than as independent application examples.

## 6. Challenges and Future Perspectives

Although previous sections (Section 4 and Section 5) have detailed the extensive applications of polypyrrole (PPy)-based coatings in areas such as surface protection, flexible sensing, electromagnetic interference shielding, and biomedicine, and have demonstrated their performance enhancements through structural design, this field still faces several fundamental and engineering challenges during the transition from laboratory research to industrial application. Based on the preceding analyses of their intrinsic structure and interfacial properties (Section 2 and Section 3), the future development of PPy-based functional coatings requires more systematic exploration in overcoming intrinsic material defects, greening processing technologies, and deeply integrating multifunctional systems.

### 6.1. Overcoming the Trade-Offs: Conductivity, Adhesion, and Stability

During the actual service of PPy-based coatings, prominent trade-offs exist among electrical conductivity, interfacial adhesion, and environmental stability, which constitute the primary physicochemical challenges in current material design. These trade-offs correspond directly to the key limitations summarized in Section 2.3, including mechanical brittleness, insufficient adhesion, conductivity decay, and long-term stability deterioration. Therefore, future research should shift from improving a single performance indicator toward coordinated regulation of conductivity, interface, mechanical robustness, and environmental durability.

First, a contradiction exists between high electrical conductivity and long-term stability. As discussed in Section 2 and Section 4, the high conductivity of PPy is heavily dependent on the oxidation state of its conjugated backbone and the presence of dopants. However, under complex aqueous environments, long-term electrochemical cycling, or washing processes, anionic dopants are prone to deintercalation, leading to irreversible conductivity decay. To address this issue, future work should focus on dopant-stabilization strategies, including macromolecular dopants with strong anchoring effects, self-doped PPy structures, and dopant-anchored cross-linked conductive networks. These approaches may suppress dopant migration and dedoping while preserving efficient charge transport. In addition, molecular design strategies such as side-chain modification and copolymerization may help improve redox stability without sacrificing the conductive pathways of PPy.

Second, the balance between interfacial adhesion and mechanical adaptability remains to be optimized. Pure PPy exhibits intrinsic brittleness due to its rigid backbone and is susceptible to microcracking under deformation. Although Section 3 and Section 4.2 indicate that introducing elastomers, such as PU and PDMS, or flexible textile skeletons can effectively alleviate stress concentration and enhance wear resistance, polymer encapsulations with low surface energy may increase interfacial contact resistance and weaken the electrical response sensitivity. Therefore, future interfacial engineering should aim to enhance adhesion, flexibility, and charge transfer simultaneously. Promising strategies include surface activation, polydopamine or silane transition layers, hydrogen bonding, coordination bonding, dynamic covalent bonding, and multilayer or bilayer coating architectures [220,221,222,223]. These designs can improve substrate bonding and mechanical damage tolerance while maintaining continuous conductive networks.

Third, structural control is essential for improving long-term coating durability. For practical applications, PPy should not be treated as an isolated conductive layer, but should be integrated into hierarchical or composite architectures that can buffer stress, block corrosive media, and stabilize the conductive/redox state. Core–shell structures, porous networks, three-dimensional skeleton-supported coatings, and hybrid systems containing two-dimensional barrier materials, corrosion inhibitors, or inorganic nanoparticles can extend diffusion pathways, reduce defect propagation, and improve service stability. Such structure-regulated strategies are particularly important for corrosion protection, flexible sensing, electrothermal coatings, and bioelectrode interfaces, where electrical function and environmental durability must be maintained simultaneously.

Finally, molecular-level structural regulation remains a fundamental pathway to overcoming performance bottlenecks. As revealed by studies on PPy derivatives and composite systems, precisely tuning the band structure of PPy through copolymerization, side-chain modification, defect engineering, and crystallinity control can fundamentally alter its charge transport dynamics and electrochemical activity. Future work should further combine molecular regulation with coating-level design, so that PPy-based coatings can achieve high conductivity, stable redox activity, strong adhesion, and improved durability at lower coating thicknesses. In this way, the intrinsic advantages of PPy can be more effectively translated into reliable coating performance under complex service conditions.

### 6.2. Towards Green Processing and Smart, Multifunctional Systems

Beyond overcoming the inherent performance trade-offs of the materials, the environmental friendliness of processing techniques and the intellectualization of application scenarios are two core directions for advancing PPy-based coatings toward practical applications. These directions are closely related to the scale-up difficulty and long-term reproducibility issues summarized in Section 2.3. Therefore, future development should not only focus on improving coating performance at the laboratory scale, but also on establishing green, reproducible, low-energy, and application-oriented manufacturing routes.

On the one hand, achieving scalable green processing techniques is imperative. Traditional chemical oxidative polymerization typically relies on large quantities of strong oxidants, such as iron(III) chloride and persulfates, and acidic solvent systems, which introduce environmental pressures and batch-to-batch inconsistencies during scale-up. Connecting with the discussion in Section 3, future process development should focus more heavily on water-based emulsions, volatile organic compound (VOC)-free conductive inks, and green synthetic routes, such as biological templating methods or the use of plant extracts as dopants. In particular, the formulation stability, storage life, viscosity control, and coating uniformity of waterborne PPy dispersions and conductive inks should be systematically optimized, because these parameters directly determine the feasibility of printing, spraying, dipping, and roll-to-roll coating processes. Furthermore, low-energy, solvent-free surface engineering technologies, such as cold plasma-assisted deposition and in situ vapor phase polymerization, provide industrially promising solutions for achieving large-area, uniform coatings on complex three-dimensional porous substrates. These methods may also reduce residual oxidants, improve coating cleanliness, and enhance interfacial adhesion, which are important for corrosion protection, wearable electronics, and biomedical coating applications. Eco-friendly corrosion-resistant composite coatings, greener manufacturing, and AI-assisted energy-management studies further suggest that process quality, environmental cost, energy efficiency, and thermal management should be jointly considered in future PPy coating scale-up [224,225,226,227,228].

On the other hand, coating systems are evolving toward highly integrated smart and multifunctional architectures. The application trends in Section 5 suggest that single protective or conductive functions can no longer meet the demands of emerging fields. Future PPy-based coating designs will increasingly emphasize stimuli-responsiveness and multiphysics coupling. For example, in energy and smart environmental control, the development of interactive interfaces integrating energy storage, Joule heating, electrochromic visual feedback, EMI shielding, and corrosion protection is critical. In biomedicine, constructing adaptive biological interfaces capable of synchronously achieving anti-infection microenvironment regulation, precise electrophysiological signal acquisition, tissue compatibility, and immunomodulation will be highly sought after. For these complex systems, PPy should be designed as an active interfacial component rather than a single conductive additive. Its conductivity, redox activity, ion transport, and structural tunability need to be coordinated with substrate mechanics, environmental stability, and device-level integration. Related visual chemical-response films and human–machine collaboration concepts may further inspire smart feedback design for wearable, biomedical, and visually responsive PPy-based interfaces [229,230].

In this context, introducing data-driven materials informatics will become a crucial tool for propelling the development of this field. Utilizing machine learning algorithms to predict the complex nonlinear relationships among polymerization conditions, dopant types, coating microstructures, and macroscopic coating properties, such as electromagnetic shielding effectiveness, corrosion resistance, adhesion strength, conductivity retention, or sensing sensitivity, is expected to accelerate the formulation optimization of multiphase composite coatings. Recent advances in multiobjective optimization and domain-adaptive learning further suggest that such data-driven approaches can help screen formulation spaces, processing windows, and application-specific constraints for PPy-based coating design [231,232,233,234]. Future studies should further establish standardized databases that include synthesis parameters, dopant chemistry, coating thickness, morphology, adhesion strength, impedance evolution, mechanical durability, and long-term stability. A multi-metric evaluation framework integrating performance, efficiency, and cost may also provide useful guidance for the industrial assessment and optimization of PPy-based multifunctional coating systems [235,236,237,238,239]. Such datasets would enable more reliable screening of dopants, fillers, interfacial layers, and processing windows for application-specific PPy-based coatings.

Overall, the future development of PPy-based coatings should combine limitation-oriented material design with scalable processing and smart system integration. Green processing strategies can reduce environmental burden and improve manufacturing reproducibility; multifunctional architecture design can expand PPy coatings from passive protection to adaptive interfacial systems; and data-driven optimization can accelerate the rational selection of formulations and coating structures. Through the integration of these strategies, PPy-based functional coatings are expected to move from laboratory-scale demonstrations toward reliable applications in corrosion protection, flexible electronics, smart sensing, energy devices, and biomedical interfaces.

## 7. Conclusions

The development of polypyrrole (PPy)-based functional coatings has driven the evolution of surface engineering from traditional passive barriers to dynamic, multi-responsive smart interfaces. Based on its unique conjugated backbone and reversible doping/dedoping chemistry, PPy provides an effective platform for integrating electrical conductivity, redox activity, and tunable morphology. As highlighted in this review, advances in fabrication strategies, ranging from electrochemical deposition to hierarchical composite design, have effectively ameliorated the inherent limitations of pure PPy, such as mechanical brittleness and structural instability. Consequently, PPy-based systems exhibit favorable performance in surface protection, including redox-mediated active anti-corrosion, mechanical wear resistance, and complex environmental purification capabilities.

Beyond traditional protective functions, the compositing of PPy with elastomers, two-dimensional nanomaterials, inorganic functional phases, and bioactive components has expanded its potential in emerging applications. The material demonstrates broad applicability in constructing lightweight electromagnetic interference (EMI) shielding networks, flexible strain and gas sensors, electrochromic energy storage devices, and immunomodulatory bioelectrodes.

Despite significant progress, the practical application of PPy-based coatings still depends on overcoming several key limitations, including mechanical brittleness, insufficient adhesion, conductivity decay, long-term instability, and scale-up difficulty. Future research should therefore shift from simply improving individual performance indicators toward integrated coating design. Composite and hybrid strategies can improve mechanical robustness and barrier performance; interface engineering can enhance substrate adhesion and charge transfer; dopant and molecular design can stabilize conductivity and redox activity; hierarchical structural control can improve multifunctional integration; and green scalable processing can promote practical manufacturing. With the integration of these strategies, PPy-based coatings are expected to evolve from laboratory-scale functional films into reliable interfacial systems for corrosion protection, flexible electronics, smart sensing, energy devices, and biomedical interfaces. Ultimately, through the deep integration of fundamental molecular engineering, green manufacturing technologies, and smart system design, PPy-based functional coatings are expected to continue playing an important role in frontier fields such as wearable electronics, sustainable environmental technologies, and advanced biomedical devices.

## Figures and Tables

**Table 1:** Comparison between PPy-based coatings and representative coating materials.

Coating Type	Main Protection/Function Mechanism	Main Limitation	Distinct Advantage of PPy-Based Coatings
Conventional organic coatings	Passive barrier against water, oxygen, and aggressive ions	Limited electrical/electrochemical functionality; local defects may cause coating failure	PPy provides both barrier protection and active redox-mediated interfacial passivation
Metallic coatings	Dense shielding, sacrificial protection, or conductive protection	High density, possible galvanic corrosion, and limited flexibility	PPy is lightweight, processable, and suitable for flexible or complex substrates
Ceramic coatings	High hardness, chemical resistance, and thermal stability	Brittleness, high-temperature processing, and poor flexibility	PPy can be prepared under mild conditions and integrated with flexible substrates
Carbon/metal filler-based conductive coatings	Conductivity mainly provided by conductive fillers	Filler aggregation, weak interfacial bonding, and limited redox activity	PPy can be polymerized in situ to form continuous conductive networks and active interfacial layers
Other conducting polymers, such as PANI and PEDOT	Conductive and electroactive behavior	PANI is strongly affected by protonation/pH; PEDOT often depends on specific monomers or formulation systems	PPy offers facile oxidative/electrochemical polymerization, broad substrate adaptability, and tunable morphology

Note: PPy = polypyrrole; PANI = polyaniline; PEDOT = poly(3,4-ethylenedioxythiophene).

**Table 2:** Main limitations of PPy-based coatings and their effects on coating performance.

Limitation	Main Origin	Effect on Coating Performance
Mechanical brittleness	Rigid conjugated backbone and limited chain mobility	Microcracking, poor flexibility, loss of coating continuity, reduced durability
Insufficient adhesion	Weak interfacial bonding, smooth substrate surface, unsuitable pretreatment, excessive coating thickness	Delamination, interfacial defects, reduced mechanical stability, accelerated electrolyte penetration
Conductivity decay	Dedoping, dopant migration, overoxidation, environmental exposure	Decreased electrical conductivity, weakened sensing/electroactive performance
Long-term stability deterioration	Electrolyte penetration, repeated redox cycling, washing, humidity, UV or chemical exposure	Reduced corrosion protection, unstable signal output, shortened service life
Scale-up difficulty	Nonuniform polymerization, batch-to-batch variation, poor dispersion stability	Inconsistent coating thickness, unstable performance, limited industrial reproducibility

## Data Availability

No new data were created or analyzed in this study. Data sharing is not applicable to this article.
